# Effects of the Ionic Liquid Structure on Porosity of Lignin-Derived Carbon Materials

**DOI:** 10.1021/acssuschemeng.3c03035

**Published:** 2023-10-07

**Authors:** Samson O. Anuchi, Kyra L. Sedransk Campbell, Jason P. Hallett

**Affiliations:** † Laboratory of Sustainable Chemical Technology, Department of Chemical Engineering, Imperial College London, South Kensington Campus, London SW7 1AZ, U.K.; ‡ Department of Chemical and Biological Engineering, University of Sheffield, Sheffield S1 3JD, U.K.

**Keywords:** lignin, slow pyrolysis, ionic liquids, ionic liquid structural properties, and lignin-derived carbons

## Abstract

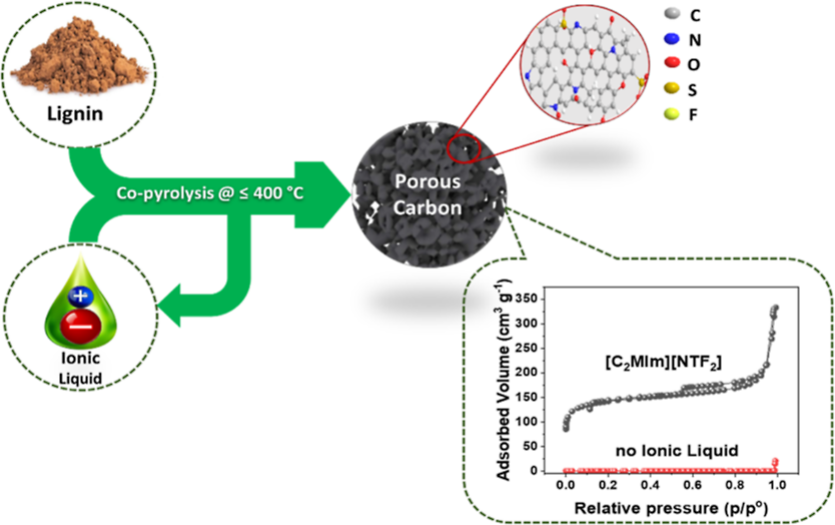

Converting lignin into advanced porous carbon materials, with desirable surface functionalities, can be challenging. While lignin-derived carbons produced by pyrolysis at >600 °C develop porosity, they also simultaneously lose nearly all their surface functional groups. By contrast, pyrolysis of lignin at lower temperatures (e.g., <400 °C) results in the formation of nonporous char that retains some surface functionalities. However, copyrolysis of lignin with some ionic liquids (ILs) at lower temperatures offers an opportunity to produce porous carbon materials with both large surface areas and an abundance of surface functional groups. This study investigates the effects of IL properties (solubility, thermal, and ionic size) on the specific surface areas of lignin-derived carbons produced by copyrolysis of lignin and ILs at 350–400 °C for 20 min. It was found that ILs that have bulky anions and small cation sizes can induce porosity in lignin-derived carbons with large surface areas. Among 16 ILs that were tested, [C_2_MIm][NTF_2_] demonstrated the best performance; the inclusion of it in the copyrolysis process resulted in lignin-derived carbons with ∼528 m^2^ g^–1^ and 0.48 cm^3^ g^–1^. Lignin-derived carbons produced using no IL, [C_2_MIm][NTF_2_], and [C_4_MIm][OTF] were further characterized for morphology, interfacial chemical, and elemental properties. The copyrolysis of lignin and [C_2_MIm][NTF_2_], and [C_4_MIm][OTF] resulted in doping of heteroatoms (N and S) on the porous carbon materials during pyrolysis reaction. The present findings contribute to a better understanding of the main property of ILs responsible for creating porosity in lignin carbon during pyrolysis.

## Abstract 2

A suitable structure for ionic liquids is crucial for the sustainable synthesis of high-specific surface area porous carbons from lignin.

## Introduction

Effective valorization of lignin is key to achieving more sustainable and competitive biorefineries.^1^ An attractive option for lignin valorization is the fabrication of porous carbon materials with desirable surface functionalities, which remains an active area of research because of increasing demand in a wide variety of applications, including adsorption, catalysis, separation, energy conversion, and storage.^2^ Lignin is an attractive renewable feedstock for carbon materials because it remains an untapped, inexpensive byproduct from paper mills and biorefineries.^3^ Also, lignin contains a higher carbon content (>60%) than other biomass feedstocks^4^ and its molecular structure resembles that of bituminous coal; as such, lignin can be considered as an alternative to totally or partially replace fossil fuel feedstocks.^4,5^

A conventional process for the production of porous carbon materials from biomass is carbonization in combination with physical or chemical activation.^4^ The carbonization regime requires high temperatures of up to 1200 °C for pore structural development, which causes the loss of the functional groups that determined its chemical properties. In contrast, low-temperature processes such as pyrolysis <400 °C or the use of compressed water, i.e., hydrothermal carbonization (170–350 °C) enables the synthesis of biochar and hydrochar,^6^ respectively. These chars are rich in functional groups but are nonporous and hence require activation steps (e.g., soft templating, hard templating, and physical and chemical activation) to develop porosity.^7,8^ In consequence, it is very difficult to simultaneously achieve both enhanced porosity and surface functionalities at improved levels on a carbon material.

The use of ILs as solvents and pore-forming agents offers an attractive approach for the synthesis of porous carbon materials at low temperature (<400 °C) with rich surface functionalities.^9^ Ionic liquids are salts that are liquids at <100 °C and are formed by the combination of an organic cation and an (in)organic anion.^10,11^ These liquid salts are termed “green” because of their very low vapor pressure (nonvolatility), which enables them to function at high temperatures.^10^ They have excellent solvating properties.^12^ Also, ILs provide different choices of cation and anion pairings, which offers an opportunity to produce different carbon nanostructures with an array of distributions of heteroatoms.^9−11^ Ionic liquids are either aprotic, containing long or short alkyl chains substituted onto imidazolium or pyridinium rings with one of several anions such as [Cl], [Br], [PF_6_], [N(CN)_2_], [BF_4_], and [CF_3_SO_3_]^13^ or protic, containing an acidic proton (H^+^) that replaces one of the alkyl groups on the cations found in aprotic ILs.^14^

Some ILs have been applied as solvents for the fractionation of lignocellulosic biomass into separate carbohydrates (cellulose and hemicellulose) and lignin streams.^10,15,16^ One economically viable IL technology, ionoSolv uses cheap protic ILs such as triethylammonium hydrogen sulfate, [TEA][HSO_4_], and *N*,*N*,*N*-dimethylbutylammonium hydrogen sulfate, [DMBA][HSO_4_] for biomass fractionation.^16^ Also, ILs have been applied as electrolytes for electrochemical storage devices (e.g., supercapacitors, lithium-ion batteries, and dye-sensitive solar cells)^17−19^ and adsorbents for CO_2_ separation and recovery processes.^20^ ILs have also been used in the fabrication of advanced porous carbon materials.^21^ Usually, ILs can function as precursors for the synthesis of carbon materials^22−25^ or advanced media or pore-forming agents for porous carbon synthesis.^8,26−29^ Both categories are commonly referred to as ionothermal carbonization (ITC). The latter category is restricted to carbohydrate sugars (e.g., glucose, fructose, and cellulose)^8,26−28^ or raw lignocellulose.^29,30^

The ITC of biomass is usually performed in an excess of IL (∼1:10 g g^–1^ biomass loading) using a pressure autoclave at 180–200 °C, which results in the addition of repolymerized volatiles and degraded ILs into the resultant carbon materials.^30,31^ Recently, Huang et al.^32^ studied the influence of the IL type on the pyrolysis of glucose and cellulose with ILs in a 1:1 g g^–1^ loading at 300 and 350 °C. They observed that ILs that moderately interact with cellulose (i.e., [C_4_MIm][OTF]), having anions with moderate hydrogen bond basicity (0.4 < β < 0.8), forms porous carbons. While ILs (e.g., [C_4_Mim][Cl]) that greatly dissolve cellulose (high β) produce nonporous carbons, because strong interaction of ILs with cellulose may decompose the IL and result in partial incorporation into the carbon product or volatilization.^32^

The ILs are not only used as the solvent but also participate in reactions involved in the carbonization as a type of catalyst more active than water in hydrothermal carbonization.^8,32−34^ The mixture of some ILs with biomass can alter their pyrolysis chemistry.^35^ These ILs are meant to have high thermal stability and good chemical properties to enable them to function as recyclable pore-forming agents for any carbon feedstock (precursor) during low-temperature pyrolysis. Some ILs can act as a catalyst to lower the pyrolytic temperature or promote dehydration, degradation, and condensation to char formation. The catalytic effects of ILs may depend on their chemical reactions with carbon precursors, which is strongly affected by the solubility of the carbon precursor in these ILs. Some ILs can simultaneously function as templates that create pore spaces within the carbon matrix.^8^ Studies have shown that some ILs having bulky anions (e.g., [C_4_MIm][NTF_2_]) form big clusters of minimal free energy within sugar carbon matrices during carbonization. The big IL clusters act as templates, creating pore spaces within the carbon matrix during carbonization.^32^

In this study, we proposed to provide mechanistic insights on the impact of IL (solvent, ionic size, and thermal) properties on copyrolysis of lignin and ILs at mild temperature conditions through specific surface areas, porosity, and structural properties of carbon materials. Therefore, experiments were based on the screening of several types of ILs for the ability to create porosity in the lignin-derived carbon nanostructure using the Brunauer–Emmet–Teller (BET) surface area as the principal indicator. Little or no ITC experimental approach on lignin is found in the literature. We initially tested the capability of 1-butyl-3-methylimidazolium, [C_4_MIm] ILs with anions ranging from [PF_6_], [NTF_2_], [OTF], [BF_4_],....... to [Cl], which differ in hydrogen bond basicity (β: 0.2–0.8). Therefore, the optimum [C_4_MIm] anion was used to screen other ILs based on the different alkyl imidazolium cationic structures, which are [C_2_MIm], [C_4_MIm], [C_6_MIm], [C_8_MIm], [C_10_MIm], and [C_4_MPyr].

## Experimental Section

### Materials

Lignin was extracted from coconut (*Cocos nucifera* L) shells by the ionoSolv process^16^ using a protic IL, [DMBA][HSO_4_] (20% H_2_O) at 170 °C for 45 min. The lignin powder was freeze-dried for 48 h before use and characterization. The moisture content of the ionoSolv shell lignin sample was 5.96 ± 0.85 wt % as determined from the mass difference after drying at 105 °C overnight. No ash content was found in the lignin samples after ashing to constant weight using a muffle oven (nabertherm + controller P 330). Bulk elemental analysis on a dry and ash free basis: C: 65.9 ± 0.35; H: 5.03 ± 0.02; N: 0.49 ± 0.04; S: 0.49 ± 0.06 and O: 28.0 ± 0.29 wt % (O = 100 – C – H – N – S, wt %). The lignin number-average molecular weight (*M*_n_) and weight-average molecular weight (*M*_w_) were 2181 ± 166 and 19,300 ± 365 g/mol, respectively. The ILs used for this study are listed in the Table S1. These ILs were purchased from Sigma-Aldrich or Iolitec and used without purification.

### Pyrolysis of Lignin-IL Mixtures

**Figure 1:**
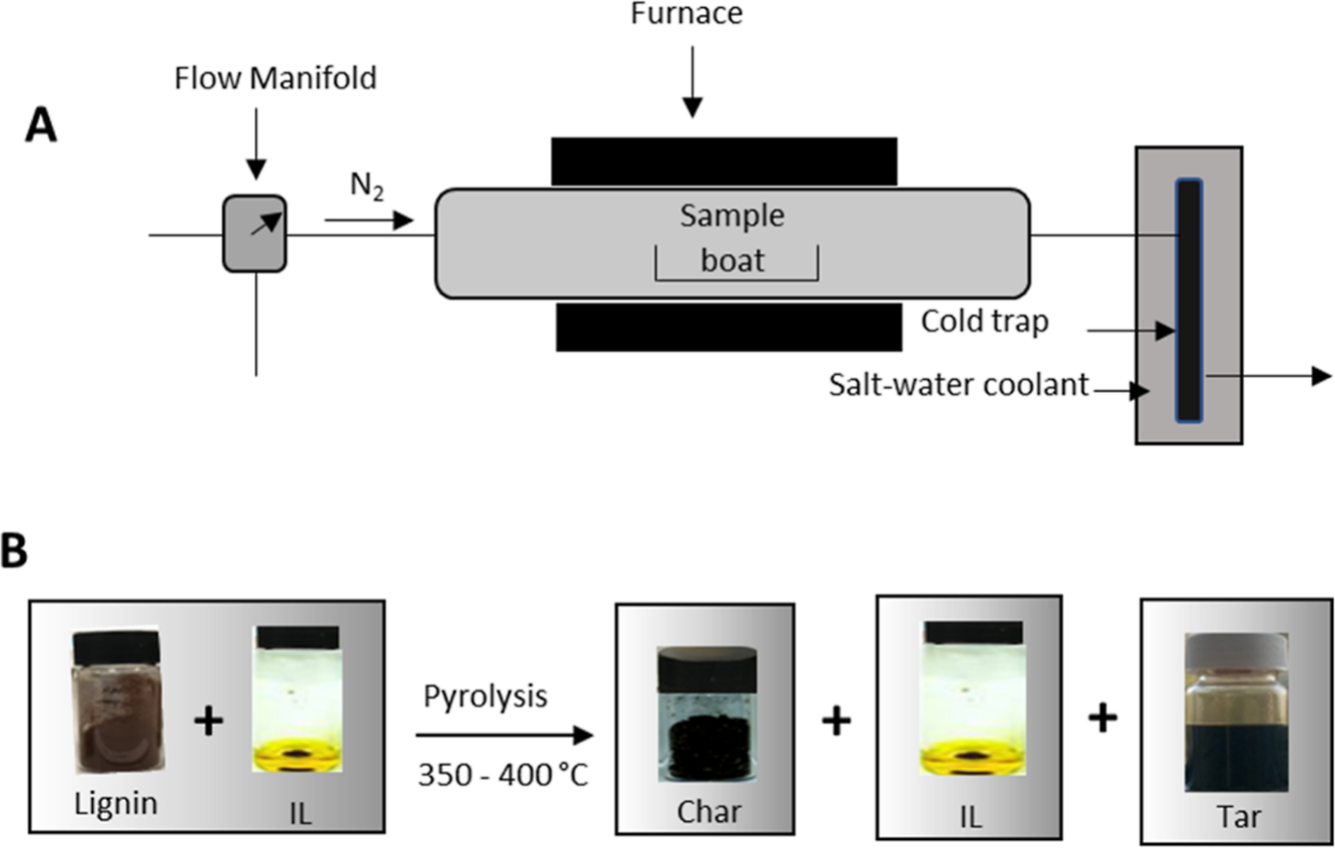
Schematic diagram of (A) pyrolysis process used in this study; (B) recovery of solid residue, IL, and tar from lignin and IL pyrolysis.

∼0.5 g each of lignin and IL were mixed and placed in a porcelain crucible. The mixture was heated under a N_2_ atmosphere (0.2 L/min) up to 350 and 400 °C (5 °C/min) for 20 min in a Lenton tubular furnace connected with a cold volatile liquid trap supported by a salt-water coolant (Figure 1). After heating, the sample was allowed to cool to room temperature. The solid residue produced was washed with ethanol using a Soxhlet overnight to thoroughly separate and recover the IL from the carbon material. The ethanol-IL was filtered through a 0.22 μm filter to remove any solid residue, and the IL was recovered and dried by rotary evaporation in a vacuum oven at 40 °C overnight. The resultant carbon was dried at 105 °C overnight before use and characterization. The condensed liquid component of the pyrolysis (tar) was recovered from the cold trap (Figure 1) after washing with methanol: chloroform (3:1), following the method described by Boot-Handford et al.^36^

### Characterization

Thermogravimetric analysis (TGA) of 10 ± 2 mg of lignin-IL mixture (1:1) was carried out using a TA Q500 TGA instrument. This analysis was performed using a Pt–Rh pan in N_2_ atmosphere (60 mL min^–1^) at 5 °C min^–1^ to 600 °C for 20 min. The thermal behavior of the mixture was estimated based on the onset (*T*_onset_) and maximum thermal decomposition temperatures (DTG_max_), and the Fourier-transform infrared spectroscopy (FT-IR) was used to investigate carbonization behavior of lignin-IL mixture and IL degradation after pyrolysis. The FT-IR spectra in the region of 4000–400 cm^–1^ was collected on a PerkinElmer Spectrum 100 spectrometer equipped with an attenuated total reflectance (ATR) cell at 4 cm^–1^ resolution.

The IL-assisted lignin-derived carbons were characterized by volumetric measurement of N_2_ at −196 °C, on a Micrometrics Tristar. The carbons were degassed at 200 °C for 3 h before the sorption measurements. Surface area and pore size distribution of the samples were determined by BET theory and a nonlocal density functional theory model (Micrometrics Instrument Corp.).

The morphology of the carbon materials was investigated by Zeiss Auriga scanning electron micrograph (SEM). X-ray photoelectron spectroscopy (XPS) was used to investigate the surface elemental compositions and the chemical states of the samples using a Thermal Fisher Nexsa equipped with a 180 hemispherical analyzer using Al Ka1 (1486.74 eV). The elemental composition and chemical states were determined after high-resolution deconvolution of each functional group (C, N, S, and O) using Thermo Avantage 5.9925 software. Carbon, hydrogen, nitrogen, and sulfur (CHNS) bulk elemental analyses were carried out in duplicates using a Vario MICRO element analyzer on an air-dried and ash-free basis. The oxygen content for each run was estimated by subtracting the sum of C, H, N, and S (wt %) from 100%.

Composition of lignin-derived tar fractions was qualitatively determined by gas chromatography mass spectrometry (GC–MS). The GC–MS analysis was conducted on a Shimadzu with the following conditions: column, Shimadzu SH-Rxi-5 ms (length: 30 m, diameter: 0.25 mm, thickness: 0.25 μm); injector temperature, 250 °C; column temperature, 40 °C (1 min), 40–300 °C (1–53 min), 300 °C (53–60 min); carrier gas, helium; flow rate, 1 mL min^–1^; emission current, 20 mA; and ionization time, 2.0 ms. Product identification was carried out by comparing the mass fragmentation patterns.

## Results and Discussion

### Ionic Liquid Selection

The quality of carbons synthesized from the copyrolysis of lignin and ILs strongly depends on the choice of ILs. Ionic liquid properties (e.g., solvation capability, thermal stability, etc.) can dictate the choice of the solvent for applications.^10,32,37^ The specific surface area of the resultant carbon was used as the principal performance indicator (Table 1). Initially, lignin was copyrolyzed with a series of 1-butyl-3-methylimidazolium [C_4_MIm]-based ILs paired with various anions (Table 2) at 350 and 400 °C. Subsequently, [NTF_2_] and [OTF] anions were paired with a range of different cations ([C_2_MIm], [C_4_MIm], [C_4_MPyr], [C_6_MIm], [C_8_MIm], and [C_10_MIm]) at 400 °C.

**Table 1:** Carbon Yield, Burn-Off, Specific Surface Areas (*S*_BET_), Total Pore Volume (*V*_T_), and IL Recovery from Co-pyrolysis of Lignin with No IL (−) and ILs, Respectively, at 350 and 400 °C for 20 min^a^

IL	temp (°C)	yield (%)	burn-off (%)	carbon yield (%)	IL recovery (%)	*S*_BET_ (m^2^ g^–^^1^)	*V*_T_ (cm^3^ g^–^^1^)
	350	72.5 ± 0.4	27.5 ± 0.4	72.5 ± 0.4		<1	0.001 ± 0.01
	400	67.4 ± 0.3	32.6 ± 0.3	67.4 ± 0.3		<1	0.001 ± 0.01
[C_4_MIm][PF_6_]	350	57.2 ± 3.6	42.8 ± 3.6	107 ± 2.8	7.3 ± 4.4	2.41 ± 0.01	0.008 ± 0.02
	400	49.9 ± 2.6	50.1 ± 2.6	88.5 ± 3.6	11.5 ± 1.6	2.32 ± 0.03	0.009 ± 0.01
[C_2_MIm][NTF_2_]	350	83.1 ± 1.0	16.9 ± 1.0	63.0 ± 0.4	103.1 ± 1.6	97.9 ± 3.21	0.243 ± 0.03
	400	72.0 ± 0.3	28.0 ± 0.3	60.2 ± 0.0	84.4 ± 0.0	527.5 ± 0.5	0.487 ± 0.04
[C_4_MIm][NTF_2_]	350	84.9 ± 1.5	15.1 ± 1.5	68.7 ± 1.0	101.2 ± 2.5	7.41 ± 0.03	0.020 ± 0.09
	400	73.0 ± 2.1	27.0 ± 2.1	65.3 ± 1.6	80.7 ± 2.6	492.5 ± 3.5	0.397 ± 0.11
[C_6_MIm][NTF_2_]	400	72.8 ± 0.9	27.2 ± 0.9	67.0 ± 0.8	78.7 ± 0.9	381.0 ± 18	0.321 ± 0.02
[C_8_MIm][NTF_2_]	400	70.8 ± 0.5	29.2 ± 0.5	60.2 ± 1.3	81.5 ± 0.4	149.5 ± 12	0.080 ± 0.12
[C_10_MIm][NTF_2_]	400	71.0 ± 0.9	29.0 ± 0.9	57.3 ± 1.0	84.7 ± 0.7	6.40 ± 0.04	0.008 ± 0.03
[C_4_MIm][OTF]	350	79.4 ± 2.3	20.6 ± 2.3	52.3 ± 7.7	106.5 ± 3.2	5.61 ± 0.61	0.004 ± 0.04
	400	68.4 ± 0.6	31.6 ± 0.6	63.2 ± 0.2	74.4 ± 0.0	389.0 ± 13	0.201 ± 0.14
[C_6_MIm][OTF]	400	69.4 ± 0.9	30.6 ± 0.9	74.1 ± 1.2	64.8 ± 0.5	172.8 ± 1.0	0.066 ± 0.01
[C_8_MIm][OTF]	400	63.4 ± 0.6	36.6 ± 0.6	69.0 ± 5.1	57.9 ± 3.9	118.8 ± 1.5	0.064 ± 0.01
[C_4_MPyr][OTF]	400	58.9 ± 9.4	41.1 ± 9.4	77.0 ± 9.3	40.9 ± 9.6	11.5 ± 0.04	0.045 ± 0.01
[C_2_MIm][OTF]	400	44.0 ± 2.9	56.0 ± 2.9	76.1 ± 1.8	11.9 ± 4.0	35.8 ± 2.13	0.025 ± 0.04
[C_4_MIm][BF_4_]	350	71.3 ± 8.6	28.7 ± 8.6	102 ± 20.1	40.0 ± 2.6	1.23 ± 0.01	0.004 ± 0.04
	400	52.7 ± 1.7	47.3 ± 1.7	71.5 ± 1.4	33.8 ± 1.8	6.84 ± 0.02	0.010 ± 0.04
[C_4_MIm][HSO_4_]	350	47.1 ± 0.0	53.2 ± 0.1	93.9 ± 0.1	0.21 ± 0.1	1.83 ± 0.01	0.004 ± 0.02
	400	45.1 ± 0.6	54.9 ± 0.6	82.9 ± 0.0	7.2 ± 1.2	10.7 ± 0.01	0.007 ± 0.01
[C_4_MIm][MeSO_4_]	350	44.6 ± 3.3	55.4 ± 3.3	85.8 ± 4.3	3.5 ± 2.3	1.90 ± 0.01	0.003 ± 0.03
	400	44.4 ± 1.3	55.6 ± 1.3	82.6 ± 3.6	6.2 ± 1.0	23.3 ± 0.01	0.004 ± 0.01
[C_4_MIm][SCN]	350	38.5 ± 0.0	61.5 ± 0.0	74.8 ± 0.7	2.1 ± 0.7	4.44 ± 0.03	0.059 ± 0.02
	400	40.2 ± 0.1	59.8 ± 0.1	75.1 ± 0.5	5.2 ± 0.3	3.50 ± 0.02	0.005 ± 0.02
[C_4_MIm][Cl]	350	35.5 ± 0.0	64.5 ± 0.0	66.4 ± 0.3	4.7 ± 0.3	4.41 ± 0.02	0.007 ± 0.02
	400	33.4 ± 1.9	66.6 ± 1.9	56.1 ± 4.0	4.0 ± 2.9	16.2 ± 0.04	0.013 ± 0.01

a Uncertainty represents standard deviation in duplicate measurements.

**Table 2:** Elemental Composition of Lignin-Derived Carbons Produced from Co-pyrolysis of Lignin with No ILs, [C_4_MIm][OTF], and [C_2_MIm][NTF_2_], Respectively, at 400 °C for 20 min^a^

lignin sample	C (%)	H (%)	N (%)	S (%)	O^b^(%)	atomic H/C	atomic O/C
no IL	70.12 ± 0.67	3.59 ± 0.20	0.23 ± 0.01	0.01 ± 0.01	26.05 ± 0.89	0.61 ± 0.03	0.28 ± 0.01
[C_4_MIm][OTF]	67.06 ± 0.02	3.32 ± 0.01	2.40 ± 0.02	0.91 ± 0.00	26.32 ± 0.01	0.59 ± 0.01	0.29 ± 0.01
[C_2_MIm][NTF_2_]	64.26 ± 0.02	3.56 ± 0.01	1.27 ± 0.03	1.18 ± 0.08	29.74 ± 0.03	0.66 ± 0.02	0.35 ± 0.02

a Uncertainty represents standard deviation from duplicate measurements.

b Calculated by difference (O = 100 – C – H – N – S, wt %).

Table 2 summarizes the key properties (yield, burnoff, surface area, and pore volume) of the synthesized, IL-assisted lignin-derived carbons; the table also reports IL recovery after copyrolysis of lignin with 16 types of ILs. The carbon yields after ethanol wash for the copyrolysis of lignin with [C_4_MIm][PF_6_] and [C_4_MIm][BF_4_] at 350 °C, respectively, were >100%. These excess masses of lignin-derived solid residues (compared to the starting lignin mass) can be attributed to the decomposed IL remaining in the pyrolyzed product. By contrast to [PF_6_] and [BF_4_], [NTF_2_] and [OTF] are regarded as significantly more stable at 350 °C. However, it was evidenced that the IL recovery range in copyrolysis at 350 °C for [C_2_MIm] and [C_4_MIm] shows that this is not universally the case. The excess IL recovery (compared to the starting IL mass) can be attributed to ethanol-soluble lignin pyrolytic products.

Following the copyrolysis of lignin and [C_4_MIm] ILs at 400 °C, only [NTF_2_] and [OTF] among the thermally stable counterparts showed IL recovery >70 wt % (Table 1). However, the IL recoveries among [NTF_2_] ILs with different alkyl chain lengths (C_2_–C_10_) are comparable. More than 84% maximum of [C_2_MIm][NTF_2_] was recovered after copyrolysis at 400 °C. ^1^H NMR and FTIR spectra for neat [C_2_MIm][NTF_2_] and recovered [C_2_MIm][NTF_2_] are comparable (Figures S1 and S2). This indicates that the reduced IL recovery can be partly due to loss of [C_2_MIm][NTF_2_] by vaporization, rather than decomposition.^38^

**Figure 2:**
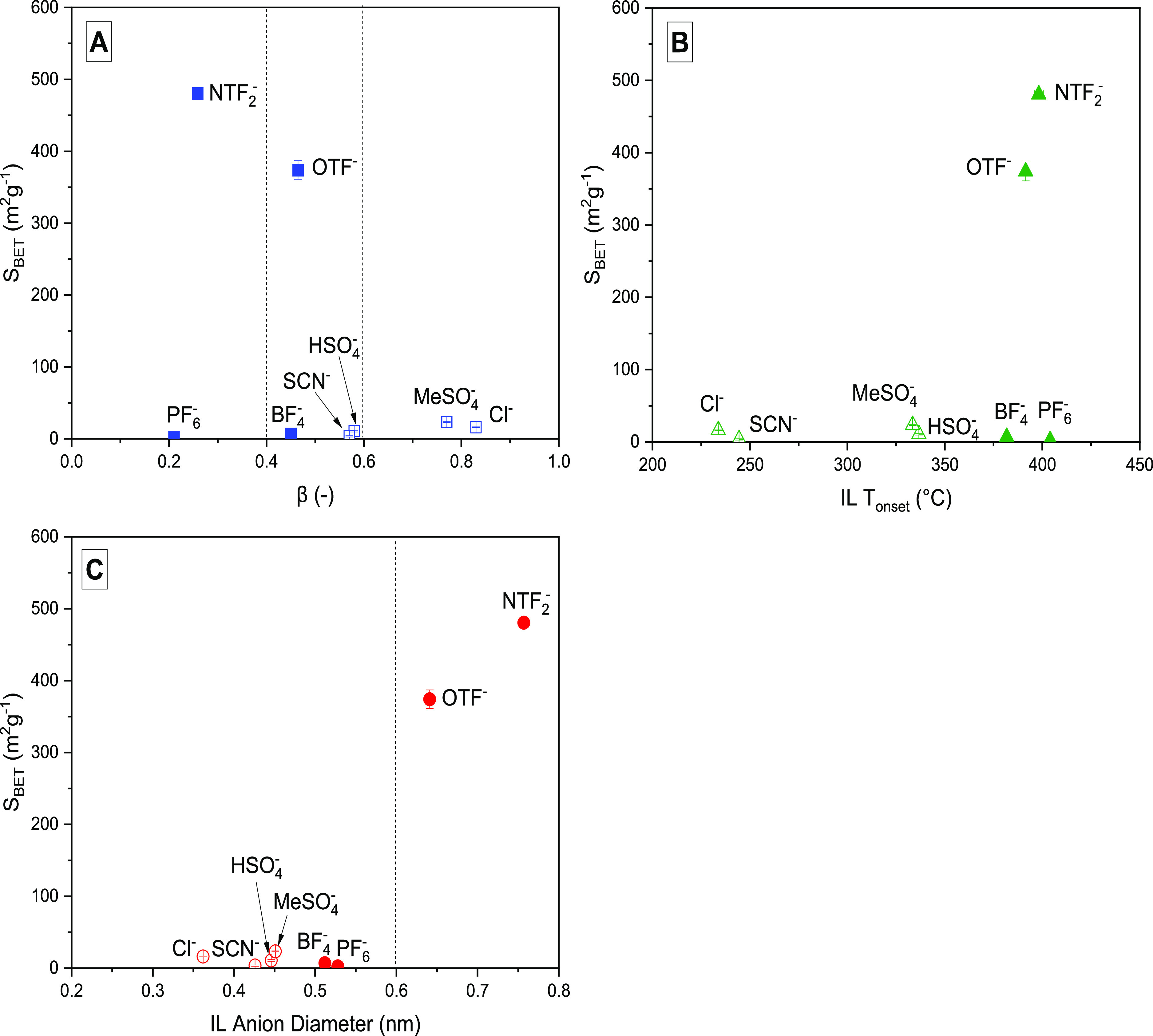
Relationship between the BET surface areas of [C_4_MIm] IL-assisted lignin-derived carbons synthesized at 400 °C and the IL properties (A) solubility by H-bond basicity (β), (B) onset temperature (*T*_onset_), and (C) anion diameter (error bars represent standard deviations from duplicate measurements of *S*_BET_ and IL *T*_onset_ as presented in Tables 1 and S3, respectively).

The relationship between key IL properties and specific surface area (*S*_BET_) of carbons, obtained after copyrolysis of lignin with different [C_4_MIm]-based ILs at 400 °C, can be observed (Figure 2). Three solvent parameters were considered: (1) Kamlet–Taft β value (hydrogen bond basicity), representing IL solvating ability; (2) IL decomposition temperature (*T*_onset_), representing thermal stability; and (3) anion diameter, representing IL size.

Within the IL experimental set, there are a range of β values from 0.21 to 0.83 (Table S2).

The solubility of lignin in ILs strongly relies on the type of IL anion.^10^ With respect to basicity, three zones can be differentiated based on their respective behavior on solubility of lignin in ILs. For β > 0.6, lignins are highly soluble; for 0.6 < β < 0.4, lignins are moderately soluble, and for β < 0.4, lignins are insoluble.^10,39−42^Figure 2A shows that highly basic ILs (β > 0.6) produced nonporous lignin-derived carbons with very low surface areas. Among the midrange basic ILs (0.6 < β < 0.4), only [C_4_MIm][OTF] generated porosity in the lignin-derived carbon with *S*_BET_ of 389 ± 13 m^2^ g^–1^. Also, [C_4_MIm][NTF_2_] generated very high surface area carbons (493 ± 3.5 m^2^ g^–1^) compared with its low-range (β < 0.4) basic IL counterpart. This establishes that no simple correlation exists between IL β and *S*_BET_ of the resulting carbon.

The copyrolysis of lignin and ILs requires that we confirm that ILs’ thermal stability determines their ability to act as pore-forming agents in lignin-derived carbon nanostructures. The IL and IL-lignin mixtures’ thermal behaviors were investigated by TGA to establish their decomposition profile and thereby estimate their decomposition temperature (*T*_onset_). The mass loss and derivative of thermal degradation (DTG) curves of the lignin, ILs, and lignin-IL mixtures are presented in Figures S5 and S6. Figure 2B reveals that [C_4_MIm] ILs containing weak nucleophilic anions (e.g., [NTF_2_], [OTF]), which are thermally more stable with *T*_onset_ > lignin DTG_max_ (Table S3), create porosity in lignin-derived carbon, by contrast to ILs with strong nucleophilic anions (e.g., [Cl], [SCN]). However, [C_4_MIm]-based containing [BF_4_] and [PF_6_] anions do not follow these classifications (i.e., strong nucleophilic anions with high *T*_onset_). This establishes that no simple correlation exists between the *T*_onset_ and *S*_BET_ of the resulting carbon.

Figure 2C demonstrates that [C_4_MIm] ILs containing anions that are <0.6 nm in diameter produced nonporous lignin-derived carbons (*S*_BET_ 2–25 m^2^ g^–1^), whereas [C_4_MIm] ILs containing anions with diameter >0.6 nm ([NTF_2_] and [OTF]) produce porous lignin-derived carbons with surface areas >380 m^2^ g^–1^. This is consistent with previous reports suggesting that bulky anions, e.g., [NTF_2_], could act as pore-forming agents during IL carbonization^22,25,37^ and in the carbonization of glucose or cellulose in ILs.^8,26,32^

Huang et al.^32^ observed that [C_4_MIm][OTF] could also act as a soft template on cellulose carbon at 350 °C, similarly to [C_4_MIm][NTF_2_], despite its smaller anion size. However, they attributed the pore-forming ability of [C_4_MIm][OTF] to be due to its chemical reaction with cellulose. Notably, IL solvating ability and thermal stability are strongly dependent on the anion size (Figure S7). That is, ILs containing anions with diameter >0.6 nm, ([NTF_2_] and [OTF]) have higher *T*_onset_ (*T*_onset_ ≥ 398 °C) and lower β values (β < 0.6). Based on these findings, it was concluded that the size of the IL anion contributes to [NTF_2_]- and [OTF]-induced porosity.

**Figure 3:**
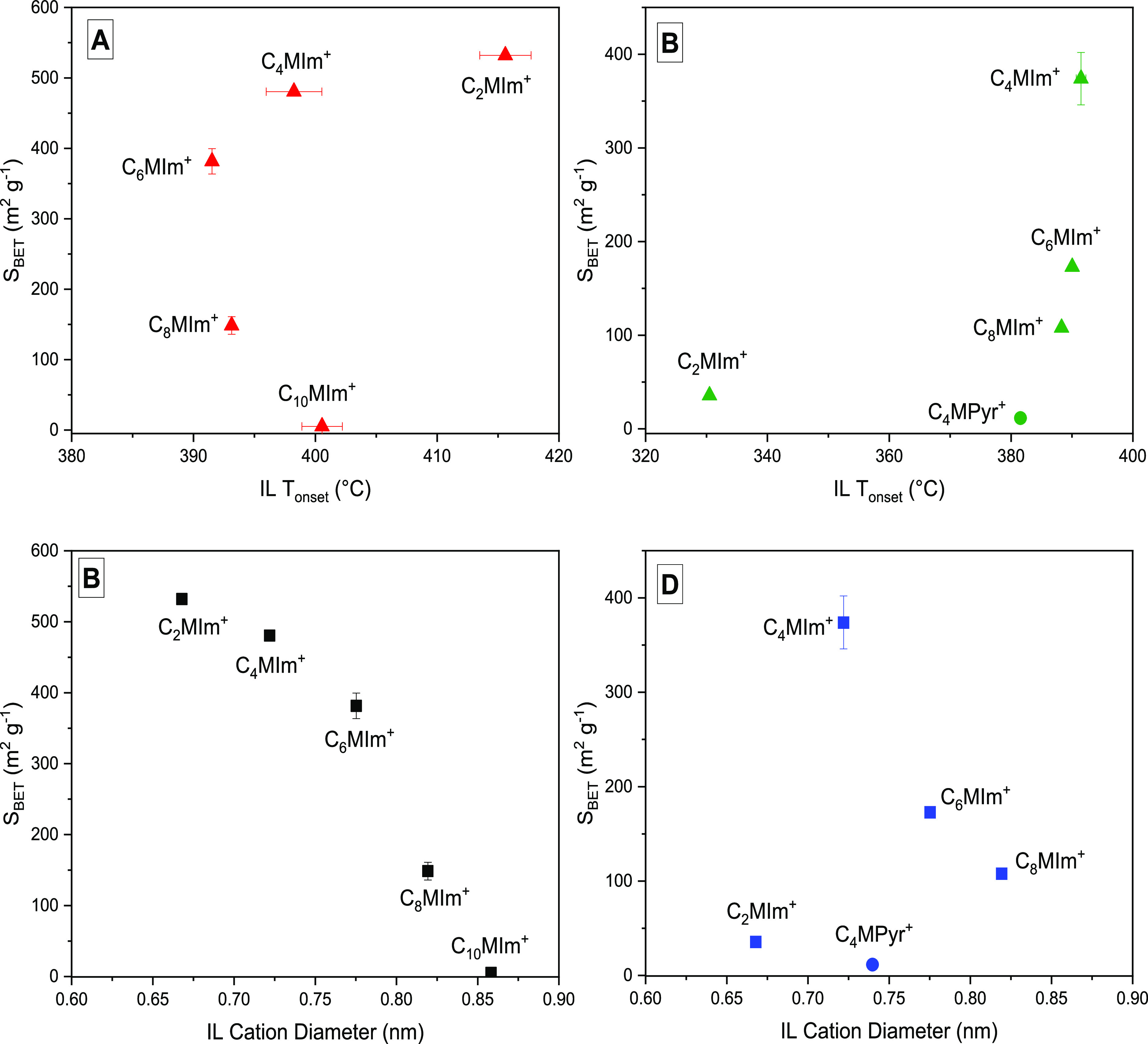
Relationship between IL onset temperature and cation diameter of (A,B) [NTF_2_]- and (C,D) [OTF]-based ILs and *S*_BET_ of the IL-assisted lignin-derived carbons synthesized at 400 °C for 20 min (error bars represent standard deviations from duplicate measurements of *S*_BET_ and IL *T*_onset_ as presented in Tables 1 and S3, respectively).

To probe further into the driver behind [NTF_2_]- and [OTF]-induced porosity, the cation was modified to imidazolium- and pyrrolidinium-based species in comparable experiments (Figure 3). In Figure 3A, the *S*_BET_ of lignin-derived carbons decreased with an increase in *T*_onset_ of [NTF_2_]-based ILs as the alkyl chain length increased from C_2_ to C_6_. The *S*_BET_ of lignin-derived carbons decreased further, despite an increase in *T*_onset_ as the alkyl chain length increased from C_6_ to C_10_.

For the case of IL cation, diameter increased with alkyl chain length from 0.67 to 0.76 nm (C_2_–C_6_), and therefore, the IL *T*_onset_ decreases (Figure S8). This indicates that for this range, ILs with smaller cation sizes have higher thermal stability, as previously reported in the literature.^11,43,44^ However, this is not universally true as observed for C_6_ to C_10_ (0.76–0.86 nm); for these larger IL cation diameters, the *T*_onset_ increased with the increasing diameter. Chancelier et al.^43^ observed a similar trend using [NTF_2_]-based ILs with different symmetric imidazolium cations containing 2 to 18 alkyl chain lengths. They suggested that the higher decomposition temperature for cations with alkyl chain <4 may be due to the highly charged imidazolium region, which prevents separation of the alkyl chains at higher temperatures. As the chain length increased from 4 to 6, a decrease in decomposition temperature was observed, because longer alkyl chains are better leaving groups, and thus formed more stable carbocations.

The *S*_BET_ of the IL-assisted lignin-derived carbons decreased rapidly from 528 to 6 m^2^ g^–1^ with the increasing IL cationic diameter, from C_2_ to C_10_ alkyl imidazolium chain lengths (0.67 to 0.86 nm), as shown in Figure 3B. The IL containing the smallest cation size, [C_2_MIm] created porosity in lignin-derived carbon with the largest *S*_BET_ (528 m^2^ g^–1^). Nonporous carbon (6 m^2^ g^–1^) was formed with [C_10_MIm], despite comparable thermal stabilities with cations of ≤4 alkyl chain. This can be ascribed to limited chemical interactions of IL containing larger cation diameters with lignin during the copyrolysis. This finding indicates that IL cation size is the major driver for the inducement of porosity in the resulting lignin-derived carbons. In essence, [NTF_2_]-based ILs containing small cation diameter <0.86 nm can create porosity with large *S*_BET_ in lignin-derived carbons.

Figure 3C shows that [OTF]-based imidazolium ILs containing different alkyl chain lengths demonstrate similar trends as [NTF_2_]-based imidazolium ILs (Figure 3A), where increasing cation diameter results in a reducing *S*_BET_. A notable exception, [C_2_MIm][OTF], creates nonporous lignin-derived carbons with the lowest *S*_BET_ (ca. 36 m^2^ g^–1^). One explanation is the very low *T*_onset_ value of [C_2_MIm][OTF] (331 °C), despite having the smallest cation size (0.67 nm) among the OTF-based imidazolium ILs (Figure 3D). Therefore, [C_2_MIm][OTF] may have decomposed completely out of the IL-lignin mixture during the pyrolysis before having maximum reaction with lignin at 400 °C, whereas [C_4_MIm] has the highest *T*_onset_, corresponding to the most stability in this set, and formed a porous structure with an *S*_BET_ of 374 m^2^ g^–1^.

The influence of IL cation size and thermal stability on porosity generation in lignin-derived carbons was further probed by comparing [C_4_Mpyr] and [C_4_MIm] (Figure 3B). The cations have similar alkyl chains but different structural characters, i.e., [C_4_Mpyr] is aliphatic and [C_4_MIm] is aromatic. The pyrolysis with [C_4_Mpyr][OTF] showed virtually no porosity; by contrast, [C_4_MIm][OTF] did (Figure 3C,D). One attribution to this difference is to the slightly lower *T*_onset_ of [C_4_Mpyr][OTF] compared with [C_4_MIm][OTF]. Previous report has shown that a fully aliphatic [C_4_Mpyr]-based IL has higher mass loss, because of easier cleavage of the alkyl chain.^45^ Therefore, [OTF]-based ILs with small cation diameter <0.8 nm can create porosity with large *S*_BET_ in lignin-derived carbons.

**Figure 4:**
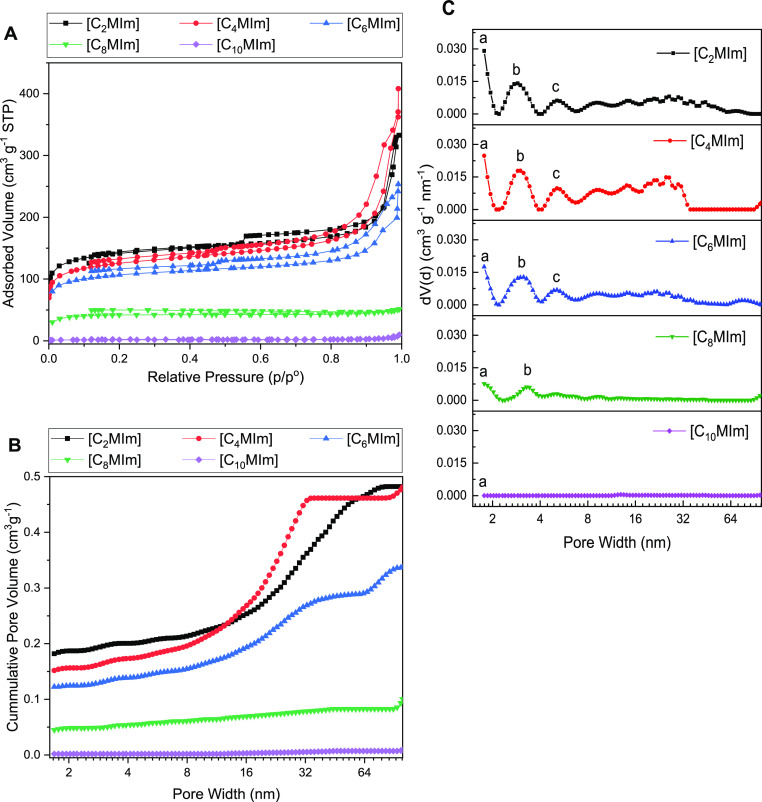
(A) Isotherm, (B) cumulative, and (C) differential pore size distributions of lignin-derived carbons produced from the copyrolysis of lignin and [NTF_2_]-based imidazolium ILs at 400 °C [pore volume indicated at (a) 1.77 nm, (b) 2.99 nm, and (c) 5.11 nm].

The porosity analyses of lignin-derived carbons produced from the copyrolysis of lignin and [NTF_2_]-based imidazolium ILs [C_*x*_MIm] are represented by both isotherms and pore size distributions (Figure 4). For C_2_–C_6_, the resulting carbons exhibit a mixture of type I and IV isotherm curves with H1 hysteresis loops^46^ (Figure 4A). By contrast, longer chain lengths C_8_ and C_10_ produced lignin-derived carbons that exhibited type I (negligible micropores) and type II (nonporous) isotherm curves, respectively (Figure 4A).

For increasing alkyl chain lengths from 2 to 6, the total pore volume of the produced lignin-derived carbons decreased from 0.49 to 0.32 cm^3^ g^–1^ (Figure 4B). The lignin-derived carbons produced using these shorter alkyl ILs have pores with significant micropore volumes at 1.77 nm (Figure 4C). The figure shows a decrease in pore volume at 1.77 nm relative to the total volume when the alkyl chain length increased. The ILs with shorter alkyl chains (<4) created higher pore volumes in lignin-derived carbons; this is explained by smaller cation size and stronger cation–anion interaction, which leads to higher thermal stabilities.^25,45^ Longer alkyl chain ILs (>4) created lower porosity in lignin-derived carbons, because their longer alkyl chain length reduces the cation–anion interaction, which causes lower thermal stability as observed with [C_6_MIm][NTF_2_] (Figure 3).^45^

However, [NTF_2_]-based imidazolium ILs with alkyl chain >6 (i.e., [C_8_MIm][NTF_2_] and [C_10_MIm][NTF_2_]) exhibited higher thermal stabilities (Figure 3). These ILs with longer alkyl chains created <0.1 cm^3^ g^–1^ total pore volume in lignin-derived carbons (Figure 4B). Figure 4C also shows initial slight changes in pore volume (relative to the total pore volume) at 2.99 and 5.11 nm as alkyl chain increased to 6 but decreases in pore volume with alkyl chain >6. This indicates that smaller IL cation size prevents agglomeration of lignin carbon particles, allowing for the formation of enlarged pores.

### GC–MS Analysis of Liquid Products of Lignin Pyrolysis with ILs

The coconut shell lignin used for this study resembles hardwood biomass, because it contains a larger proportion of syringyl (3,5-dimethoxy-4-hydroxyphenyl, S) than guaiacyl (4-hydroxy-3-methoxyphenyl, G) subunits after characterization, which results in a S/G ratio of 1.5.^47^ Nonetheless, these subunits are linked together various types of aryl ether (C–O, e.g., β-O-4) and condensed (C–C) linkages.^10,35^ There is no general consensus on the lignin pyrolysis mechanism; it is widely proposed that lignin pyrolysis involves a two-step mechanism: primary and secondary reactions.^35,48^ An alternative suggestion of an intermediate lignin pyrolysis reaction has also been proposed.^21,49,50^

**Figure 5:**
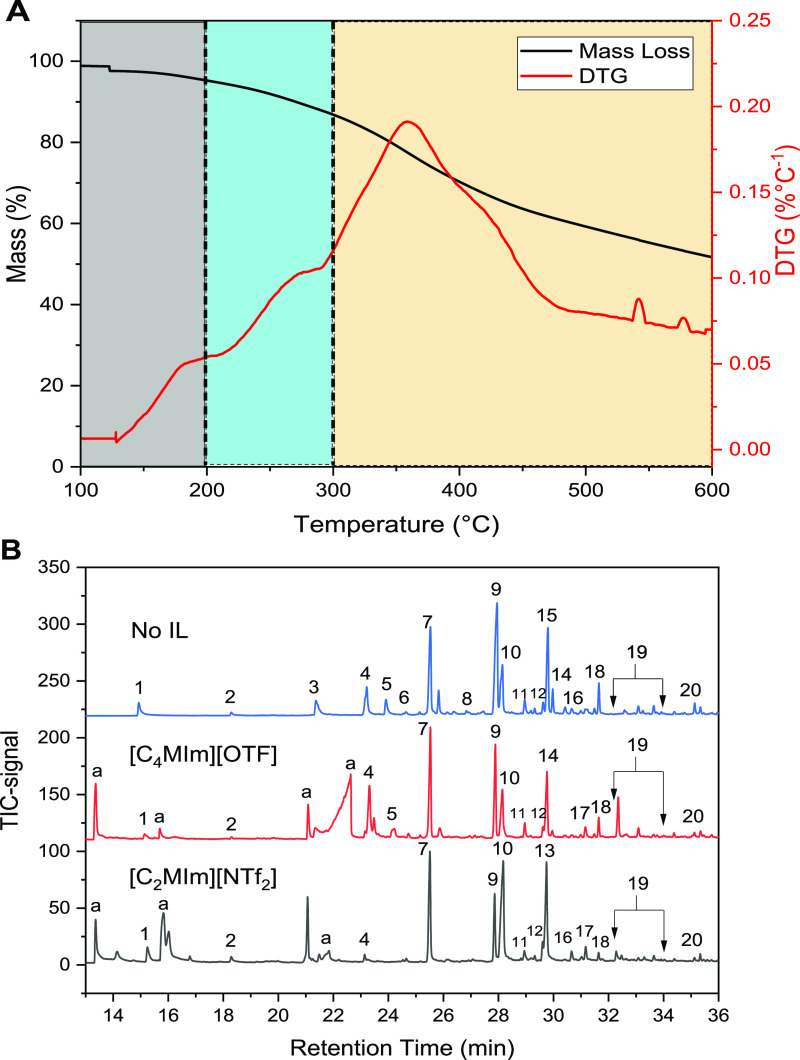
(A) Mass loss (%) and DTG (% °C^–1^) curves of the shell lignin using TGA between 100 and 600 °C for 20 min at a heating rate of 5 °C min^–1^, where 100–200 °C (highlighted in gray), 200–300 °C (highlighted in blue), and 300–600 °C (highlighted in yellow) represent pyrolytic regions under 40 mL/min N_2_ flow. This in addition to mass loss (%) and DTG (% °C^–1^) under air at 600–1000 °C (25 °C min^–1^). (B) GC–MS chromatograms of volatile liquid products from the pyrolysis of lignin without ILs (no IL) and with ILs ([C_4_MIm][OTF] and [C_2_MIm][NTF_2_]) at 400 °C (5 °C min^–1^) for 20 min. Some selected peaks are assigned to (1) phenol, (2) 2-methoxyphenol, (3) pyrocatechol, (4) 3-methoxycatechol, (5) 4-methylcatechol, (6) 5-methoxycresol, (7) 2,6-dimethoxyphenol, (8) vanillin, (9) 3,5-dimethoxycresol, (10) carbomethoxy phenol, (11) acetovanillone, (12) vanillic acid, (13) homovanillic acid, (14) 2-guaiacylacetone, (15) toluene, (16) 4-vinylsyringol, (18) 1-guaiacylacetone, (19) *E*-4-propenylsyringol (compound eluted at this region at a retention time of between 32 and 34 min), and (20) syringlacetone (^a^) Imidazole.

The TGA curve of the shell lignin under N_2_ from 100 to 600 °C (Figure 5A) displayed similar trends to those from evolved gas mass spectrometry^21^ and thermal carbon analysis.^51^ The DTG curve provides a summary of the lignin decomposition across 100 to 600 °C, which can be separated into three continuous regions, 100–200, 200–300, and 300–600 °C. Primary reactions of lignin pyrolysis have been found to occur within the first and second region, which involves the breakage of α-O-4 and β-O-4 linkages, resulting in the release of phenolic monomers and lightweight gases (H_2_ and CO_2_) as well as conversion to phenolic oligomers.^21^ Li et al.^21^ proposed that guaiacol-type compounds: 4-vinylphenol and 2-methoxy-4-vinylphenol are the major phenolic product from the primary pyrolysis reaction of lignin, while other phenolics such as phenol, 2-methoxyphenol (guaiacol), 1,2-dihyroxyphenol (catechol), ethylphenol, 2-methoxy-4-propenylphenol and isoeugenol, and vanillin are generated during the secondary reaction. Studies have also shown that secondary pyrolytic reactions of lignin occur within 300–600 °C (third region) with DTG of 374 °C, with a higher number of free radicals than lower temperature regions.^21,52^

Figure 5B shows the GC–MS chromatograms of liquid products (tar) generated after the pyrolysis of lignin with and without ILs ([C_2_MIm][NTF_2_] and [C_4_MIm][OTF]) at 400 °C, while the full GC–MS chromatograms and summary of the list of compounds from the tar products identified by the GC–MS are presented in Figure S9. The overall yields of lignin tar acquired after pyrolysis are 2.28 ± 0.03% (no IL), 1.94 ± 0.01% ([C_4_MIm][OTF]), and 1.33 ± < 0.01% ([C_2_MIm][NTF_2_]). The chromatograms of the tars (Figure 5B) show the presence of a typical lignin primary tar component, 4-vinylsyringol (16) from the S-lignin subunit, along with phenol (1), 2-methoxyphenol (2), 2,6-dimethylphenol (7), vanillin (8), acetovanillone (11), vanillic acid (12), homovanillic acid (13), 2-guaiacylacetone (18) syringlacetone (20), *E*-4-propenylsyringol (19), pyrocatechol (3), 3-methoxycatechol (4), 4-methylcatechol (5), and 5-methoxycresol (6), which may have been formed from the intermediate and secondary reactions. Studies have shown that secondary reaction of lignin pyrolysis proceeds with homolytic cleavage of the O–CH_3_ bonds on the lignin aromatic ring. This reaction leads to the conversion of O–CH_3_ to –OH, –CH_3_, –H, thus forming catechol, cresol, and phenols, respectively.^21,35,48,49^

The occurrence total ion chromatography peak intensities of phenol, cresol, and catechol differ significantly among tar products from the pyrolysis of lignin with no IL, [C_4_MIm][OTF], and [C_2_MIm][NTF_2_] (Figure 5B). Phenol (1), pyrocatechol (3), 3-methoxycatechol (4), 4-methylcatechol (5), and 5-methoxycresol (6) were found in tar produced from the pyrolysis of lignin without ILs. These species were also observed in the tars produced from pyrolysis of lignin with ILs. Phenol, 3-methoxycatechol, and 4-methylcatechol were found in the lignin tar produced with [C_4_MIm][OTF], whereas only phenol and 3-methoxycatechol eluted from lignin tar produced with [C_2_MIm][NTF_2_] (Figure 5B). The figure shows that peaks belonging to phenol, 3-methoxycatechol, and 4-methylcatechol for the lignin tars produced with [C_4_MIm][OTF], [C_2_MIm][NTF_2_], and without ILs are different (no IL > [C_4_MIm][OTF] ≫ [C_2_MIm][NTF_2_]). This result demonstrates the influence of aprotic solvents ([C_4_MIm][OTF] and [C_2_MIm][NTF_2_]) on the pyrolysis of lignin at temperatures below ≤400 °C. Kotake et al.^53^ showed that the addition of aprotic solvents (e.g., diphenoxybenzene) to lignin can suppress the polymerization reactions during pyrolysis.

### Characterization of Lignin-Derived Solid Residues

#### Morphology

**Figure 6:**
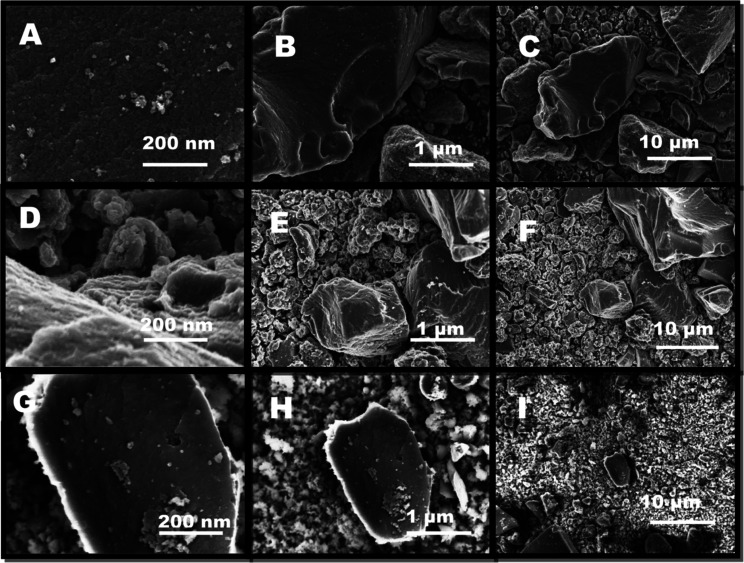
SEMs of lignin-derived carbons from copyrolysis of lignin with no IL (A–C) with [C_4_MIm][OTF] (D–F), and [C_2_MIm][NTF_2_] (G–I), respectively, at 400 °C for 20 min.

SEM analysis was used to investigate the structure of the lignin-derived carbons produced from the pyrolysis of lignin: (1) without IL (no IL), (2) with [C_4_MIm][OTF], and (3) with [C_2_MIm][NTF_2_]. Imaging, by SEM, taken post-pyrolysis, with no added IL, show an apparently dense structure, with micron-scale particles (Figure 6A–C). The mixture of lignin with [C_4_MIm][OTF] resulted in overall smaller particle sizes (submicron), although some greater than 1 μm were observed. The appearance of these particles is distinct from those without ILs as the same smoothness is not observed. The imaging shows that particle have no apparent consistent nature or shape (Figure 6D–F). Mixing lignin with [C_2_MIm][NTF_2_] resulted in the smallest particle sizes, as compared to the addition of the [OTF] or no IL (Figure 6G–I).

#### Elemental and Chemical Composition

The experimentally measured bulk elemental properties (CHNS and O) of lignin-derived carbons, prepared from the pyrolysis of lignin with (1) no IL, (2) [C_4_MIm][OTF], and (3) [C_2_MIm][NTF_2_], are presented in Table 2. Individual elemental compositions are reported, where some distinctions are seen. The H/C ratio, which estimates the degree of aromaticity and stability of the carbons, and the O/C ratio, which indicates the abundance of oxygen functional groups and polarity of the carbons,^21^ are similar for the untreated (no IL) and [C_4_MIm][OTF]-treated case. By contrast, treatment with [C_2_MIm][NTF_2_] have larger H/C and O/C ratios (Table 2).

**Figure 7:**
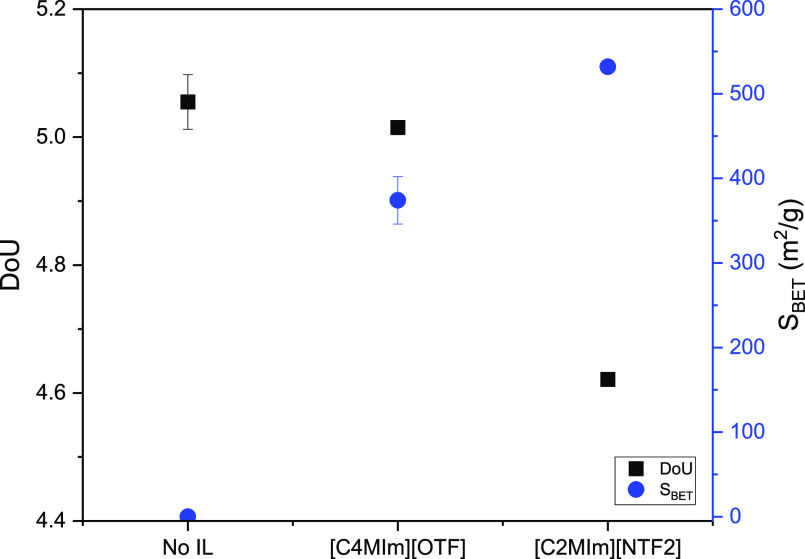
DOU and specific surface area (*S*_BET_) of lignin-derived carbons produced from copyrolysis of lignin with no IL, [C_4_MIm][OTF], and [C_2_MIm][NTF_2_], respectively at 400 °C for 20 min (error bars represent standard deviations from duplicate measurements).

The lower values of the H/C and O/C, as seen for the untreated lignin and the [C_4_MIm][OTF]-treated lignin, indicate that these have both a higher degree of aromaticity and degree of unsaturation (DOU) than the [NTF_2_] case. The DOU, which measures the carbonization extent, decreases for carbons derived from the copyrolysis of lignin with [C_2_MIm][NTF_2_], respectively (Figure 7). The intercalation of the [NTF_2_] IL (small cations and bulky anions) into the lignin carbon matrix suppresses the agglomeration of carbon particles, leading to enhanced porosity.

Table 2 also highlights the increase in nitrogen and sulfur on the carbons after copyrolysis of lignin with [C_4_MIm][OTF] and [C_2_MIm][NTF_2_], respectively. The excess nitrogen and sulfur have leached onto the lignin-derived carbons ([C_4_MIm][OTF]) by partial decomposition of [C4MIm][OTF]. In contrast, after ethanol wash, [C_2_MIm][NTF_2_] remained in the carbon residue, suggesting that the N and S content increased, since the structural properties remained unchanged based on the FTIR and ^1^H NMR spectra (Figures S1 and S2).

**Figure 8:**
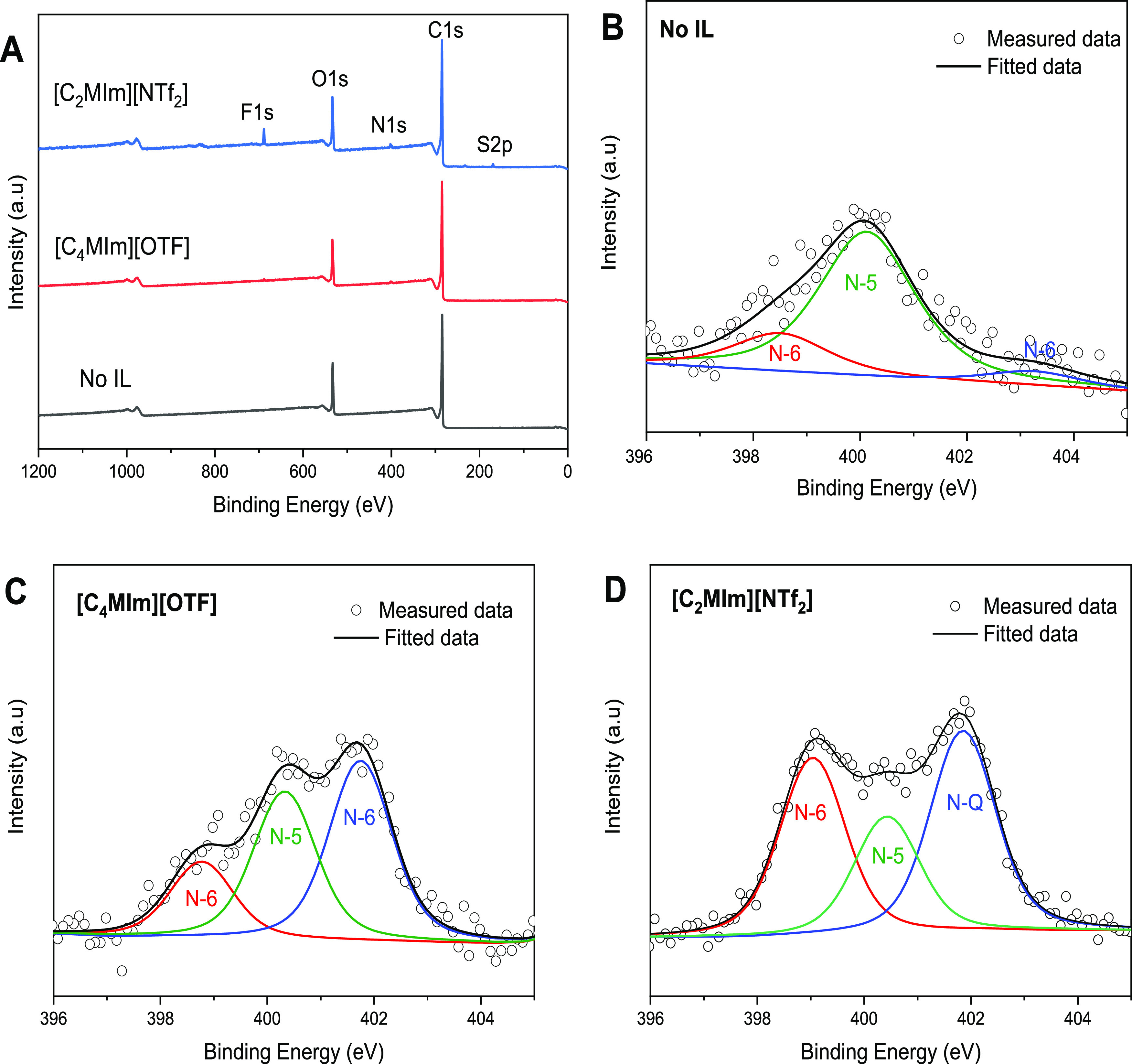
X-ray photoelectron spectroscopy analysis of lignin-derived carbons. (A) Survey spectra of lignin-derived carbons. N 1s deconvolution of lignin-derived carbons produced from copyrolysis of lignin with (B) no IL, (C) [C_4_MIm][OTF], and (D) [C_2_MIm][NTF_2_], respectively, at 400 °C for 20 min.

The XPS survey spectra, as well as the atomic composition and chemical states of heteroatoms, in lignin-derived carbons produced from pyrolysis of lignin: (1) without IL (no IL), (2) with [C_4_MIm][OTF], and (3) with [C_2_MIm][NTF_2_], are presented in Figure 7A and Table 3. There is a notable difference in carbon and oxygen content from the XPS analysis to the bulk elemental results (Table 2). These differences arise from XPS sensitivity to surface functionalities on the carbon solids. Using deconvolution, three distinct nitrogen groups were identified, including pyrrolic-N (400.0 eV), pyridinic-N (398.5 eV), and quaternary-N (402.8 eV), which were all observed in the lignin-derived carbon samples (Figure 8B–D). Previous studies have suggested that a combination of pyridinic and pyrrolic nitrogen groups contributes more to carbon electrochemical activity than other nitrogen species.^54,55^ After examination of the F 1s and S 2p core-level spectra, lignin-derived carbon ([C_2_MIm][NTF_2_]) displayed the highest fluorine and sulfur content, which can partly be due to residual IL, [C_2_MIm][NTF_2_] trapped in the carbon material that was not completely removed by ethanol-wash after pyrolysis. These heteroatoms, particularly sulfur, have been shown to improve the electronic reactivity of the porous carbons through modification of the electronic structure and charge distribution of the carbon atoms.^54,55^

**Table 3:** XPS Surface Elemental Composition of Lignin-Derived Carbons Produced from Co-pyrolysis of Lignin with No IL, [C_4_MIm][OTF], and [C_2_MIm][NTF_2_], Respectively, at 400 °C for 20 min (Single Measurement)

atomic composition (%)							
sample	C 1s	S 2p	O 1s	F 1s	N-6 (pyridinic)	N-5 (pyrrolic)	N-Q (quaternary)
no IL	80.6	0.1	18.5	NA	0.11	0.43	0.04
[C_4_MIm][OTF]	83.0	0.2	15.5	0.31	0.18	0.35	0.42
[C_2_MIm][NTF_2_]	77.9	2.0	15.8	2.57	0.66	0.44	0.75

#### Structural Properties

**Figure 9:**
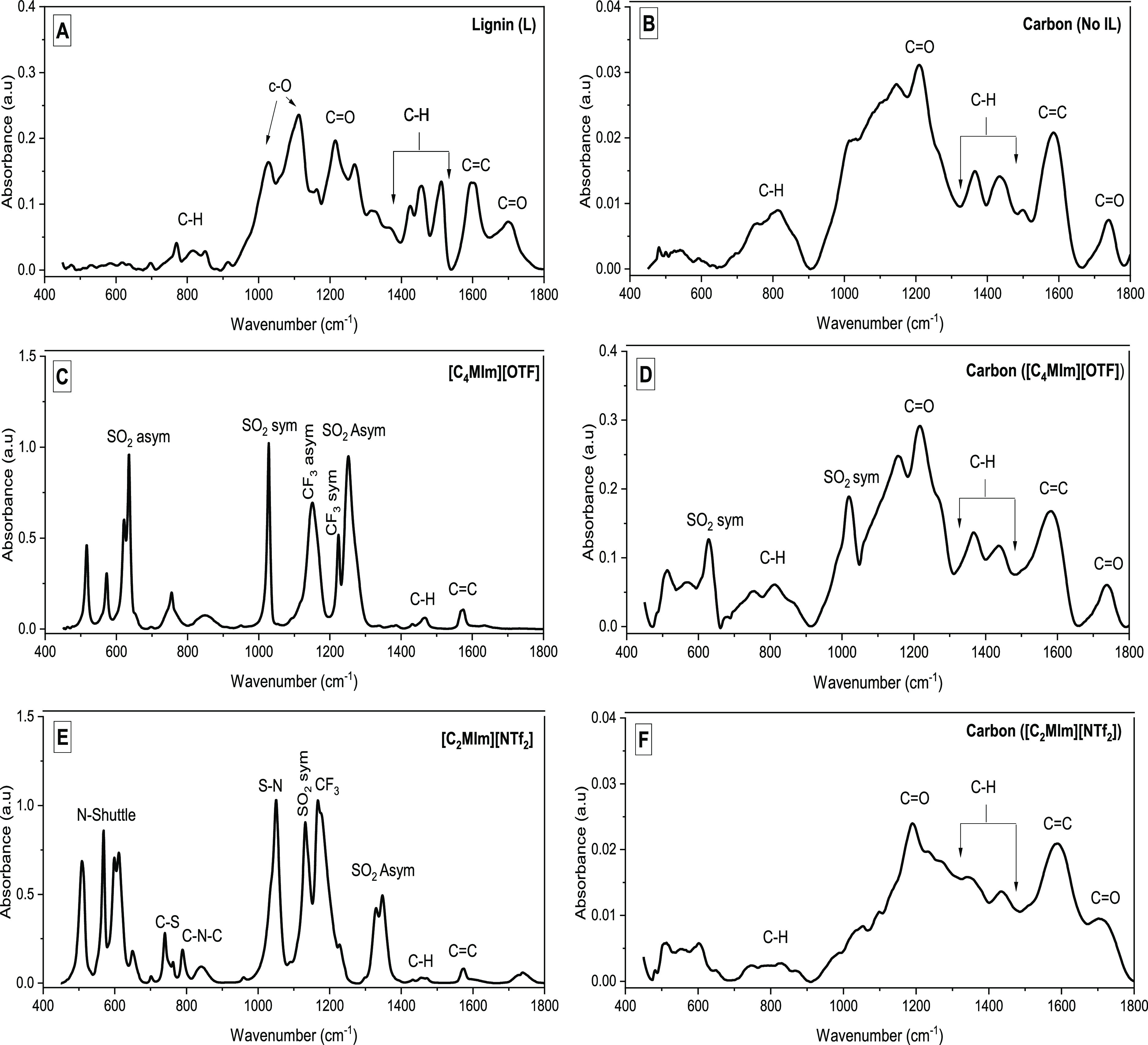
FTIR spectra of lignin (A), lignin-derived carbon produced with no IL (B), [C_4_MIm][OTF] (C), lignin-derived carbon produced with [C_4_MIm][OTF] (D), [C_2_MIm][NTF_2_] (E), and lignin-derived carbon produced with [C_2_MIm][NTF_2_] (F) (all carbons were produced at 400 °C for 20 min).

The FT-IR analysis helps us understand how the ILs ([C_4_MIm][OTF] and [C_2_MIm][NTF_2_]) affect the chemical functionalities of the lignin-derived carbons. The spectra of [C_2_MIm][NTF_2_] and [C_4_MIm][OTF] show distinct absorption peaks at 830, 1450, and 1570 cm^–1^ for C–N–C, alkyl, and C=C groups, respectively, of the [C_2_MIm]^+^ and [C_4_MIm]^+^ cations (Figures 9, S10), while the characteristic peaks of the [NTF_2_]^−^ anions observed at 600, 1050, 1120, 1190, and 1350 cm^–1^ represent N_shuttle_, S–N, symmetric SO_2_, CF_3_, and asymmetric SO_2_, respectively.^25^ As for the [OTF]^−^ anion, distinct peaks at 640, 1030, 1190, 1200, and 1280 cm^–1^ represent asymmetric SO_2_, symmetric SO_2_, asymmetric CF_3_, symmetric CF_3_, and asymmetric SO_2_, respectively (Figure 9).

In Figure 9, FT-IR spectra of carbon derived from lignin produced following pyrolysis in the absence of ILs (no IL) resemble the spectra of that produced with ILs ([C_2_MIm][NTF2] and [C4MIm][OTF]). The carbon ([C_4_MIm][OTF]) spectrum showed two peaks at 640 and 1030 cm^–1^, which resemble SO_2_ peaks observed in [C_4_MIm][OTF], suggesting deposition of the IL pyrolysate on the lignin-derived carbon, which were not completely removed by ethanol-wash after pyrolysis.

## Conclusions

The conversion of lignin to porous carbons that have both enhanced porosity, large surface areas, and rich surface functionalities can be challenging. The reason is that high-temperature pyrolysis >600 °C usually result in the loss of surface functionalities, despite causing large surface area and porosity on the carbon material. On the other hand, low-temperature pyrolysis <400 °C adversely affords the formation of porosity, but the resulting carbon materials have richer surface functionalities. Using IL-assisted pyrolysis of lignin opens a pathway to creating high porous carbon materials, which have both large surface areas and rich surface functional groups. We developed a novel strategy for preparing IL-assisted lignin-derived porous carbons through the copyrolysis of lignin and ILs at a temperature below ≤400 °C. Among the key IL properties studied against the *S*_BET_ of lignin-derived carbons, only the ionic sizes were observed to drive the formation of porosity in the lignin carbon nanostructures.

To produce large surface area lignin-derived carbons, the IL is required to have bulky anions and small cation sizes. Therefore, [C_2_MIm][NTF_2_] was selected to provide the best performance among 16 ILs investigated based on the resultant carbon-specific surface area. Co-pyrolysis of lignin and [C_2_MIm][NTF_2_] at 400 °C produced lignin-derived carbons, which have a large surface area exceeding 500 m^2^ g^–1^, more than 500 times higher than that produced without ILs. Lignin-derived carbons produced using [C_2_MIm][NTF_2_] and [C_4_MIm][OTF] show highly functional surface groups (e.g., N, O, S, and F) when compared to counterparts without ILs.

## Supporting Information Available

Properties of lignin used for copyrolysis experiments; ionic sizes in diameter and Kamlet Taft solubility parameters of selected ILs; ^1^H NMR and FTIR spectra of neat and recycled [C_2_MIm][NTF_2_] and [C_2_MIm][OTF] obtained from copyrolysis of lignin with [C_2_MIm][NTF_2_] and [C_2_MIm][OTF], respectively, at 400 °C for 20 min; isotherms and pore-size distributions of lignin-derived carbons produced from copyrolysis of lignin with no IL and [C_4_MIm] ILs (containing [PF_6_], [NTF_2_], [OTF], [BF_4_], [HSO_4_], [MeSO_4_], [SCN], and [Cl]), respectively, at 350 and 400 °C for 20 min; weight loss and thermal degradation (DTG) of ILs and lignin-IL mixtures, respectively, at 25–600 °C under N_2_ flow; thermal stability indicators (*T*_start_, *T*_onset_, and DTG_max_) of lignin, ILs, and lignin-IL mixtures; relationship between IL β, *T*_onset_, and anion diameter; relationship between IL *T*_onset_, *S*_BET_ of lignin-derived carbons, and IL cation size; GC chromatograms and distribution products of tars produced from copyrolysis of lignin with no IL, [C_4_MIm][OTF], and [C_2_MIm][NTF_2_], respectively, at 400 °C, and FT-IR spectra of lignin, [C_4_MIm][OTF], [C_2_MIm][NTF_2_], lignin-derived carbons produced from copyrolysis of lignin with no IL, C_4_MIm][OTF], and [C_2_MIm][NTF_2_], respectively, at 400 °C (PDF)
