# Supplementary material for: Effects of the Ionic Liquid Structure on Porosity of Lignin-Derived Carbon Materials
Source: ACS Sustain Chem Eng. 2023 Oct 7;11(42):15228–41. doi: 10.1021/acssuschemeng.3c03035 (PMC10598883; doi:10.1021/acssuschemeng.3c03035)
Supplement: Supplementary file 1 — sc3c03035_si_001.pdf [file sc3c03035_si_001.pdf]

Supporting Information

Effects of Ionic Liquid Structure on Porosity of Lignin-derived carbon materials

Samson O. Anuchi[a], Kyra L. Sedransk Campbell[b] and Jason P. Hallett[a*]

[a]Laboratory of Sustainable Chemical Technology, Department of Chemical Engineering, Imperial College London, South Kensington Campus, London, SW7 1AZ, United Kingdom.

[b]Department of Chemical and Biological Engineering, University of Sheffield, Sheffield, S1 3JD, United Kingdom.

* Corresponding author

Email address: j.hallett@imperial.ac.uk

Total number of pages: 14

Total number of figures: 10

Total number of tables: 4

Total number of figures: 10

Figure S1. ^{1}H NMR spectra of neat and recycled [C_2MIm][NTF_2] and [C_2MIm][OTF] obtained from co-pyrolysis of lignin with [C_2MIm][NTF_2] and [C_2MIm][OTF], respectively at 400 °C for 20 minutes.

Figure S2. FT-IR spectra of neat and recycled [C_2MIm][NTf_2] and [C_2MIm][OTF] obtained from co-pyrolysis of lignin with [C_2MIm][NTF_2] and [C_2MIm][OTF], respectively at 400 °C for 20 minutes.

Figure S3. (A) isotherms, (B) cumulative PSD and (C) differential PSD of lignin-derived carbons produced from co-pyrolysis of lignin with no IL and [C_4MIm] ILs (containing [PF_6], [NTF_2], [OTF], [BF_4], [HSO_4], [$MeSO_4$], [SCN], and [Cl]), respectively at 350 °C for 20 minutes.

Figure S4. (A) isotherms, (B) cumulative PSD and (C) differential PSD of lignin-derived carbons produced from co-pyrolysis of lignin with no IL and [C_4MIm] ILs (containing [PF_6], [NTF_2], [OTF], [BF_4], [HSO_4], [$MeSO_4$], [SCN], and [Cl]), respectively at 400 °C for 20 minutes.

Figure S5. Curves of weight loss and derivative of thermal degradation (DTG) of (A – B) [C4MIm]-based ILs, (C – D) [NTF_2]-based ILs, and (E – F) [OTF]-based ILs, respectively under N_2 gas at 25 - 600 °C.

Figure S6. Curves of weight loss and DTG of (A – B) lignin, mixture of lignin and [C_4MIm]-based ILs, (C – D) mixture of lignin and [NTF_2]-based ILs, and (E – F) mixture of lignin and [OTF]-based ILs, respectively under N_2 gas at 25 - 600 °C.

Figure S7. Relationship between β, T_{onset} and anion size (diameter) of ionic liquids.

Figure S8. Relationship between IL T_{onset} , BET surface areas of lignin-derived carbons and IL cation size (diameter).

Figure S9. GC Chromatograms of tars produced from co-pyrolysis of lignin with (A) no IL, (B) [C_4MIm][OTF], and (C) [C_2MIm][NTF_2], respectively at 400 °C for 20 minutes.

Figure S10. FT-IR spectra of lignin, [C_4MIm][OTF], [C_2MIm][NTF_2], lignin-derived carbons produced from co-pyrolysis of lignin with no IL, C_4MIm][OTF], and [C_2MIm][NTF_2], respectively at 400 °C for 20 minutes.

Total number of tables: 4

Table S1. Properties of lignin used for co-pyrolysis experiments.

Table S2. Key Properties (ionic size in diameter and Kamlet Taft Solubility parameters) of Selected Ionic Liquids.

Table S3. T_{start}, $T_{onset,}$ and DTG_{max} of lignin, mixture of lignin and ionic liquids estimated from the weight loss and DTG curves.

Table S4. Distribution of tar products produced from co-pyrolysis of lignin with (A) no IL, (B) [C_4MIm][OTF], and (C) [C_2MIm][NTF_2], respectively at 400 °C for 20 minutes.

Table S1. Properties of lignin used for co-pyrolysis experiments (lignin was isolated from the ionoSolv pretreatment of coconut shell with [DMBA][HSO_4]-water mixture at 170 °C for 45 minutes, as described by Anuchi *et al.*[1]).

Elemental Analysis (dry and ash free)	Composition (wt.%)
C	65.9 ± 0.35
H	5.03 ± 0.02
N	0.49 ± 0.04
S	0.49 ± 0.06
O[a]	28.0 ± 0.29
**Molecular Weight**	**MW (g mol^{-1})**
M_n	2181 ± 166
M_w	19300 ± 365

[a]Calculated by difference (O = 100 – C – H – N – S, wt. %). Molecular weight parameters, M_n M_w represent number of average molecular weight and weight average molecular weight , respectively.

Table S2. Key Properties (ionic size in diameter and Kamlet Taft Solubility parameters) of Selected Ionic Liquids.[2–5]

IL	Cation	Anion	D Cation (nm)	D Anion (nm)	$\pi*$	α	β	β -α
[C_4MIm][PF_6]	1-butyl-3-methylimidazolium	hexafluorophosphate	0.72	0.53	1.03	0.63	0.21	-0.42
[C_2MIm][NTF_2]	1-ethyl-3-methylimidazolium	bis(trifluoromethanesulfonyl) imide	0.67	0.76	0.91	0.67	0.29	-0.38
[C_4MIm][NTF_2]	1-butyl-3-methylimidazolium	bis(trifluoromethanesulfonyl) imide	0.72	0.76	0.91	0.62	0.26	-0.37
[C_6MIm][NTF_2]	1-hexyl-3-methylimidazolium	bis(trifluoromethanesulfonyl) imide	0.78	0.76	0.98	0.65	0.25	-0.40
[C_8MIm][NTF_2]	1-methyl-3-octyllimidazolium	bis(trifluoromethanesulfonyl) imide	0.82	0.76	0.97	0.60	0.28	-0.32
[C_{10}MIm][NTF_2]	1-decyl-3-methylimidazolium	bis(trifluoromethanesulfonyl) imide	0.86	0.76	NA	NA	NA	NA
[C_4MIm][OTF]	1-butyl-3-methylimidazolium	trifluoromethanesulfonate	0.72	0.64	1.00	0.63	0.46	-0.17
[C_8MIm][OTF]	1-methyl-3-octyllimidazolium	trifluoromethanesulfonate	0.72	0.64	0.97	0.60	0.59	-0.01
[C_4MPyrr][OTF]	1-butyl-1-methylpyrrolidinium	trifluoromethanesulfonate	0.74	0.64	1.02	0.40	0.46	0.07
[C_2MIm][OTF]	1-ethyl-3-methylimidazolium	trifluoromethanesulfonate	0.67	0.64	1.18	NA	0.40	NA
[C_4MIm][BF_4]	1-butyl-3-methylimidazolium	tetrafluoroborate	0.72	0.51	0.98	0.67	0.45	-0.21
[C_4MIm][HSO_4]	1-butyl-3-methylimidazolium	hydrogen sulfate	0.72	0.45	NA	0.60	0.58	-0.02
[C_4MIm][$MeSO_4$]	1-butyl-3-methylimidazolium	methanesulfonate	0.72	0.45	1.02	0.44	0.77	0.33
[C_4MIm][SCN]	1-butyl-3-methylimidazolium	thiocyanate	0.72	0.43	1.08	NA	0.57	NA
[C_4MIm][Cl]	1-butyl-3-methylimidazolium	chloride	0.72	0.36	1.03	0.49	0.83	0.34

NA: not available.

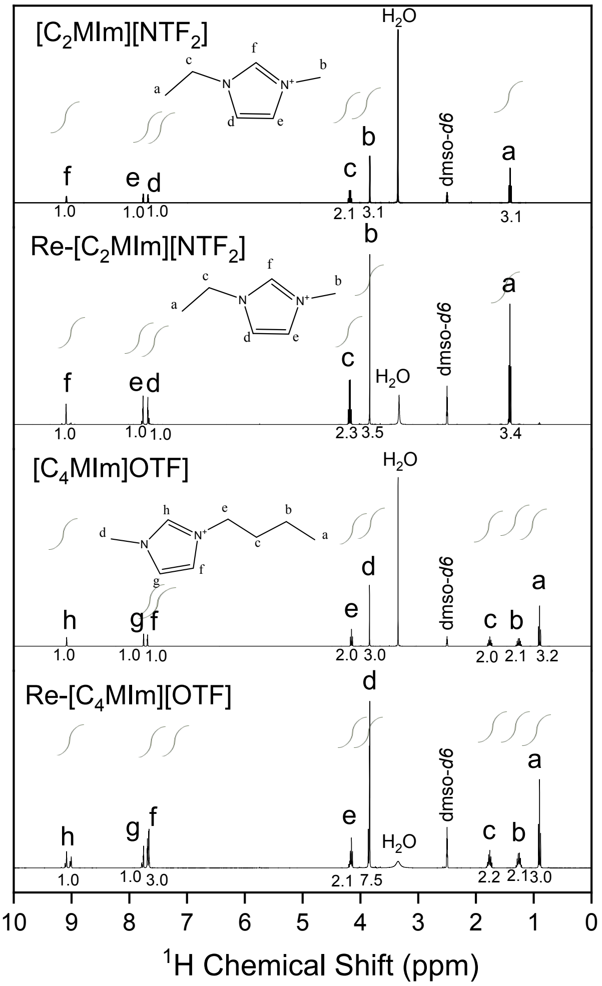

Figure S1. ^{1}H NMR spectra of neat and recycled [C$_2$MIm][NTF$_2$] and [C$_2$MIm][OTF] obtained from co-pyrolysis of lignin with [C$_2$MIm][NTF$_2$] and [C$_2$MIm][OTF], respectively at 400 °C for 20 minutes.

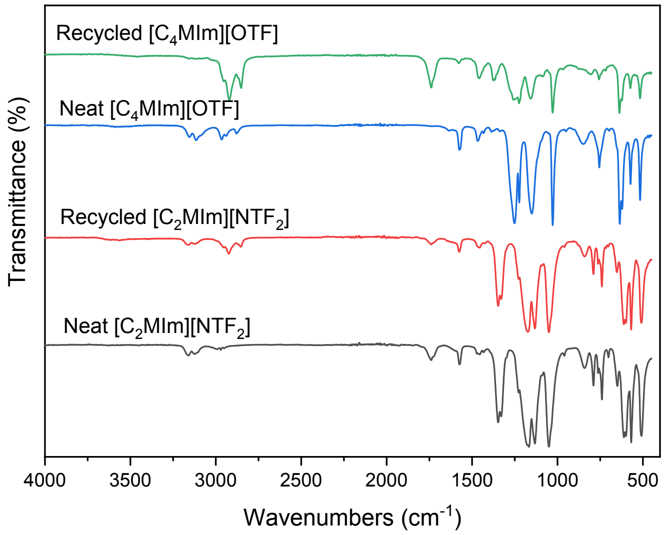

Figure S2. FT-IR spectra of neat and recycled [C_2MIm][NTf_2] and [C_2MIm][OTF] obtained from co-pyrolysis of lignin with [C_2MIm][NTF_2] and [C_2MIm][OTF], respectively at 400 °C for 20 minutes.

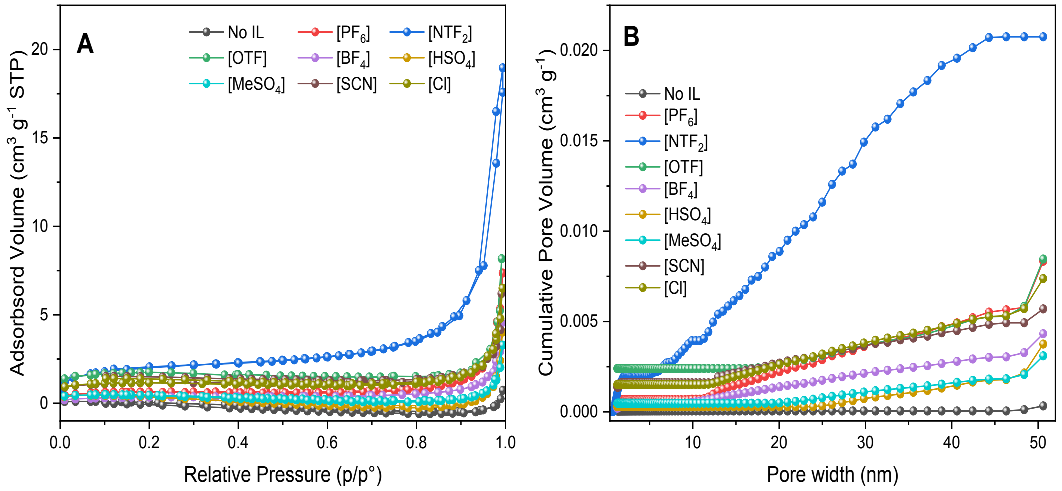

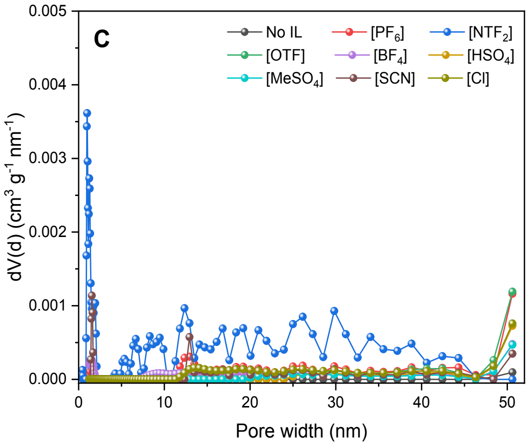

Figure S3. **(A)** isotherms, **(B)** cumulative PSD and **(C)** differential PSD of lignin-derived carbons produced from co-pyrolysis of lignin with no IL and [C_4MIm] ILs (containing [PF_6], [NTF_2], [OTF], [BF_4], [HSO_4], [$MeSO_4$], [SCN], and [Cl]), respectively at 350 °C for 20 minutes.

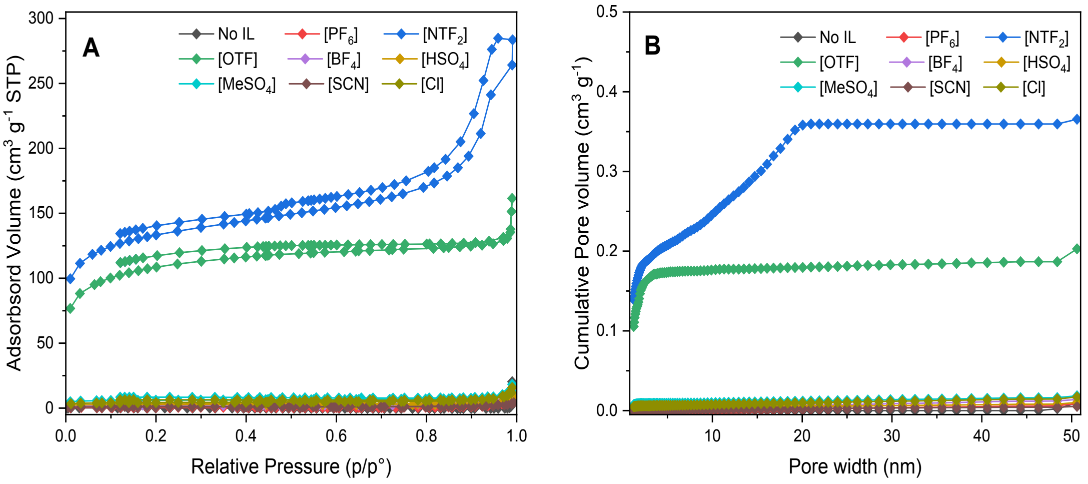

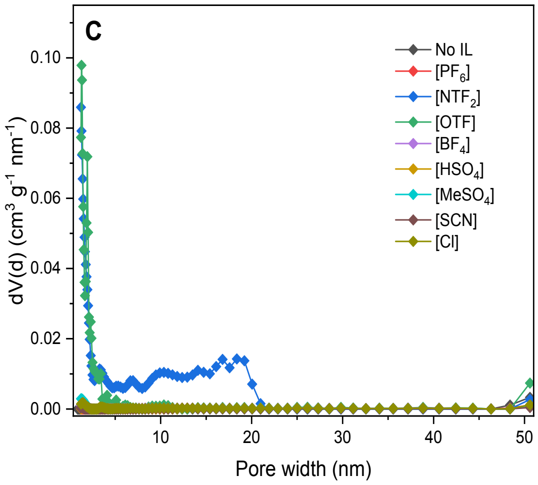

Figure S4. (A) isotherms, **(B)** cumulative PSD and **(C)** differential PSD of lignin-derived carbons produced from co-pyrolysis of lignin with no IL and $[C_4MIm]$ ILs (containing $[PF_6]$, $[NTF_2]$, [OTF], $[BF_4]$, $[HSO_4]$, $[MeSO_4]$, [SCN], and [Cl]), respectively at 400 °C for 20 minutes.

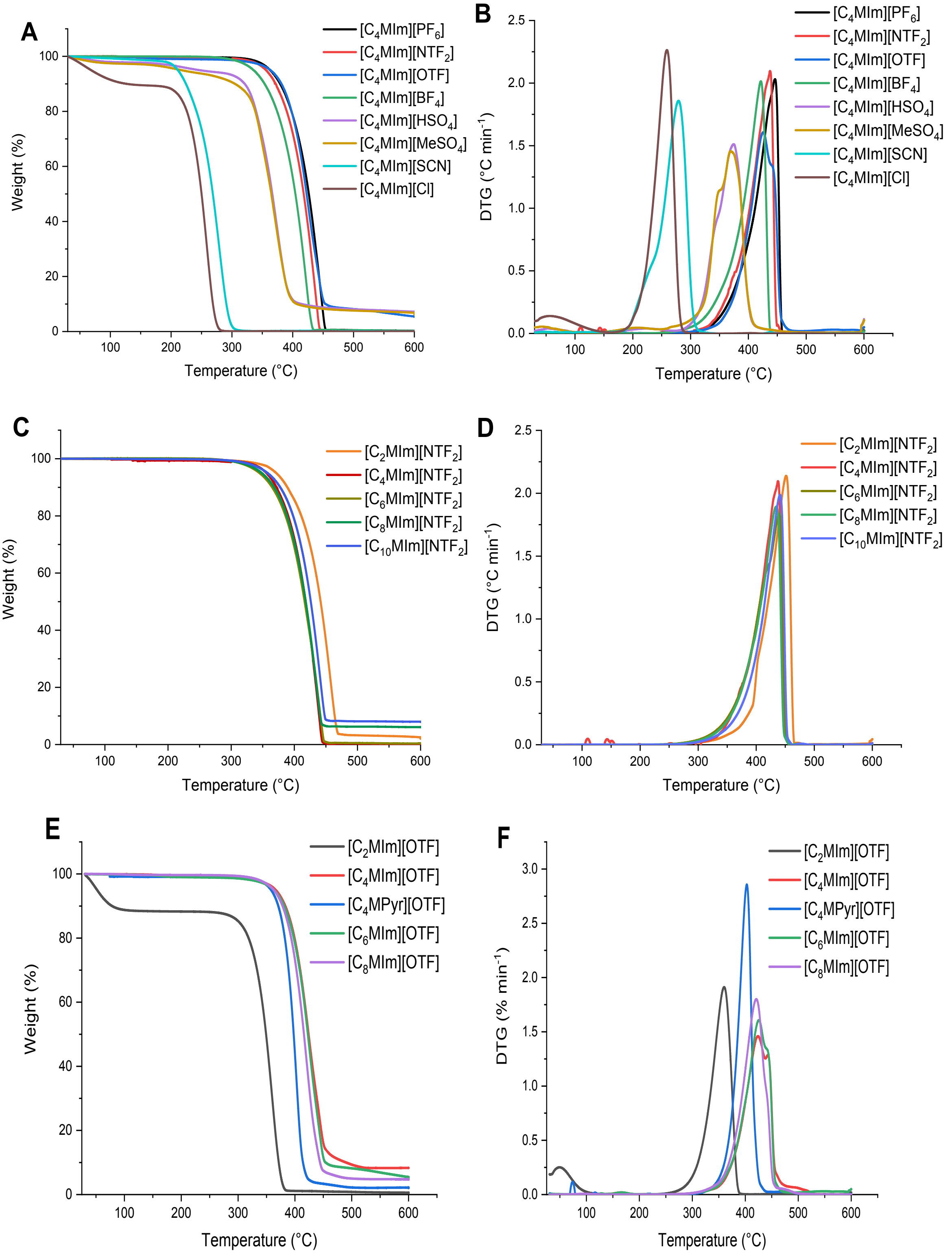

Figure S5. Curves of weight loss and derivative of thermal degradation (DTG) of **(A – B)** [C$_4$MIm]-based ILs, **(C – D)** [NTF$_2$]-based ILs, and **(E – F)** [OTF]-based ILs, respectively under N_2 gas at 25 - 600 °C.

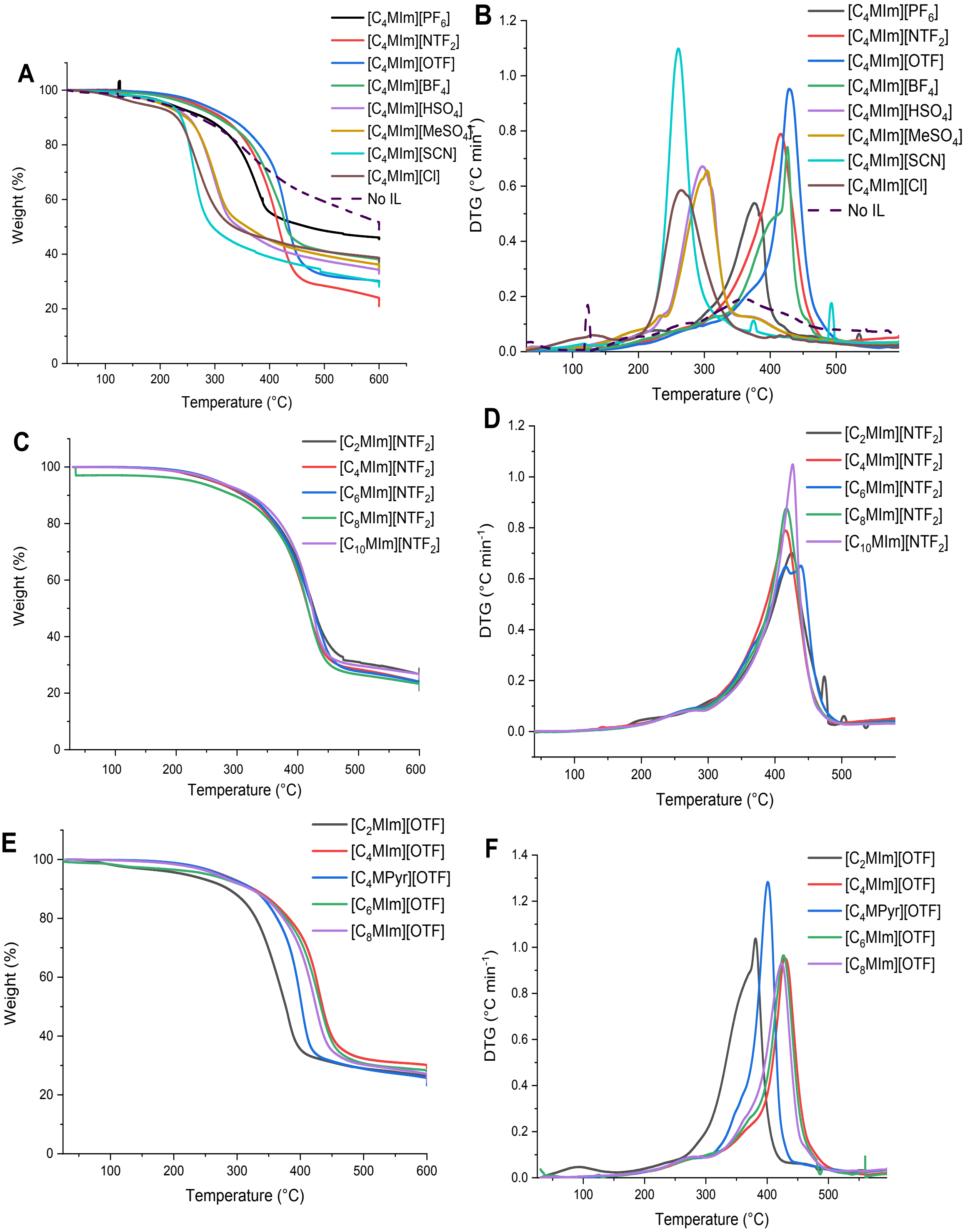

Figure S6. Curves of weight loss and DTG of **(A – B)** lignin, mixture of lignin and [C_4MIm]-based ILs, **(C – D)** mixture of lignin and [NTF_2]-based ILs, and **(E – F)** mixture of lignin and [OTF]-based ILs, respectively under N_2 gas at 25 - 600 °C.

Table S3. T_{start}, $T_{onset,}$ and DTG_{max} of lignin, mixture of lignin and ionic liquids estimated from the weight loss and DTG curves.

	Ionic Liquid (IL)			Lignin and IL Mixture		
	**T_{Start} (°C)**	**T Onset (°C)**	**DTG (°C)**	**T Start (°C)**	**T Onset (°C)**	**DTG (°C)**
**Lignin**	-	-	-	205 ± 0.01	271 ± 0.11	374 ± 0.01
**$[C_4MIm][PF_6]$**	362 ± 0.01	404 ± 0.01	446 ± 0.02	216 ± 0.01	320 ± 0.01	376 ± 0.01
**$[C_2MIm][NTF_2]$**	372 ± 0.21	416 ± 2.11	454 ± 2.91	258 ± 2.59	356 ± 2.30	424 ± 1.25
**$[C_4MIm][NTF_2]$**	354 ± 2.05	398 ± 2.30	439 ± 1.81	266 ± 2.78	352 ± 0.21	415 ± 1.30
**$[C_6MIm][NTF_2]$**	345 ± 0.01	392 ± 0.01	439 ± 0.01	267 ± 1.65	341 ± 5.41	438 ± 1.01
**$[C_8MIm][NTF_2]$**	348 ± 0.01	393 ± 0.01	436 ± 1.32	262 ± 0.04	358 ± 3.76	412 ± 5.01
**$[C_{10}MIm][NTF_2]$**	358 ± 0.01	401 ± 1.72	442 ± 1.01	267 ± 0.50	374 ± 0.79	425 ± 1.36
**$[C_2MIm][OTF]$**	298 ± 0.02	331 ± 0.04	360 ± 0.01	259 ± 0.01	326 ± 0.01	381 ± 0.04
**$[C_4MIm][OTF]$**	363 ± 0.01	392 ± 1.01	425 ± 0.80	272 ± 3.29	382 ± 1.91	428 ± 1.20
**$[C_4MPyr][OTF]$**	358 ± 0.02	382 ± 0.01	403 ± 0.01	270 ± 2.51	361 ± 2.79	399 ± 2.05
**$[C_6MIm][OTF]$**	360 ± 0.02	390 ± 0.07	422 ± 0.50	269 ± 0.04	379 ± 0.01	426 ± 0.05
**$[C_8MIm][OTF]$**	362 ± 0.59	388 ± 0.20	420 ± 0.10	267 ± 0.20	375 ± 3.21	425 ± 0.94
**$[C_4MIm][BF_4]$**	336 ± 0.11	382 ± 0.05	422 ± 0.05	251 ± 0.89	350 ± 8.69	417 ± 8.69
**$[C_4MIm][HSO_4]$**	246 ± 0.01	337 ± 0.01	375 ± 0.01	205 ± 0.20	254 ± 1.11	297 ± 0.10
**$[C_4MIm][MeSO_4]$**	216 ± 0.02	334 ± 0.01	369 ± 0.01	202 ± 0.01	255 ± 0.15	304 ± 0.21
**$[C_4MIm][SCN]$**	211 ± 0.04	245 ± 0.02	279 ± 0.01	224 ±1.91	237 ± 0.09	261 ± 0.12
**$[C_4MIm][Cl]$**	68.3 ± 0.01	234 ± 0.03	259 ± 0.03	166 ± 0.01	229 ± 0.01	265 ± 0.01

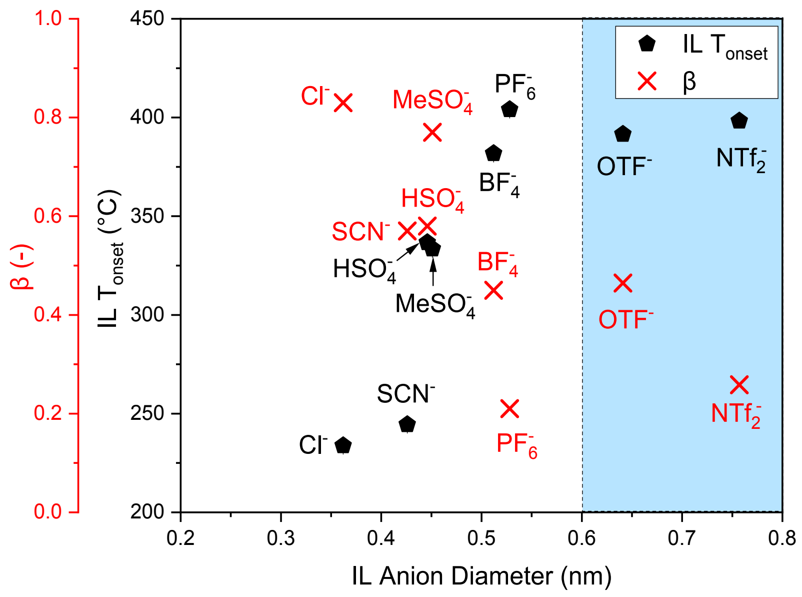

Figure S7. Relationship between β, T_{onset} and anion size (diameter) of ionic liquids.

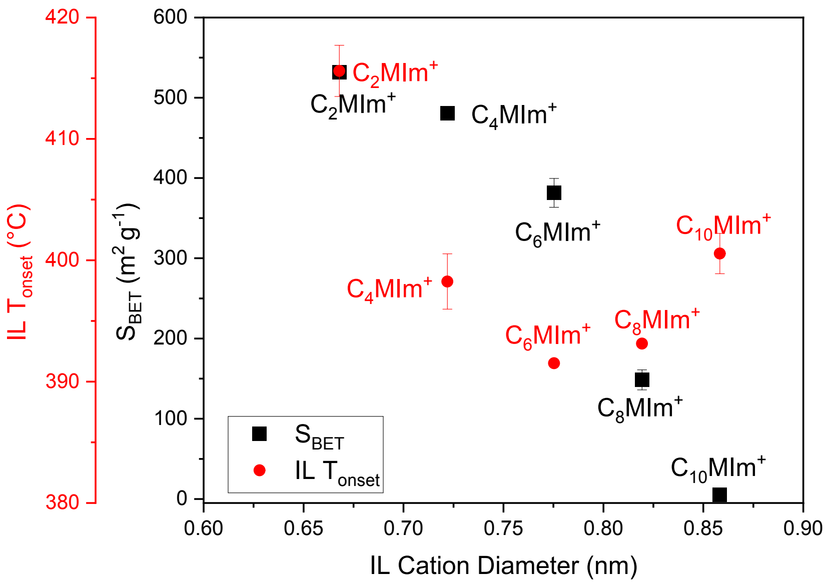

Figure S8. Relationship between IL T_{onset} , BET surface areas of lignin-derived carbons and IL cation size (diameter).

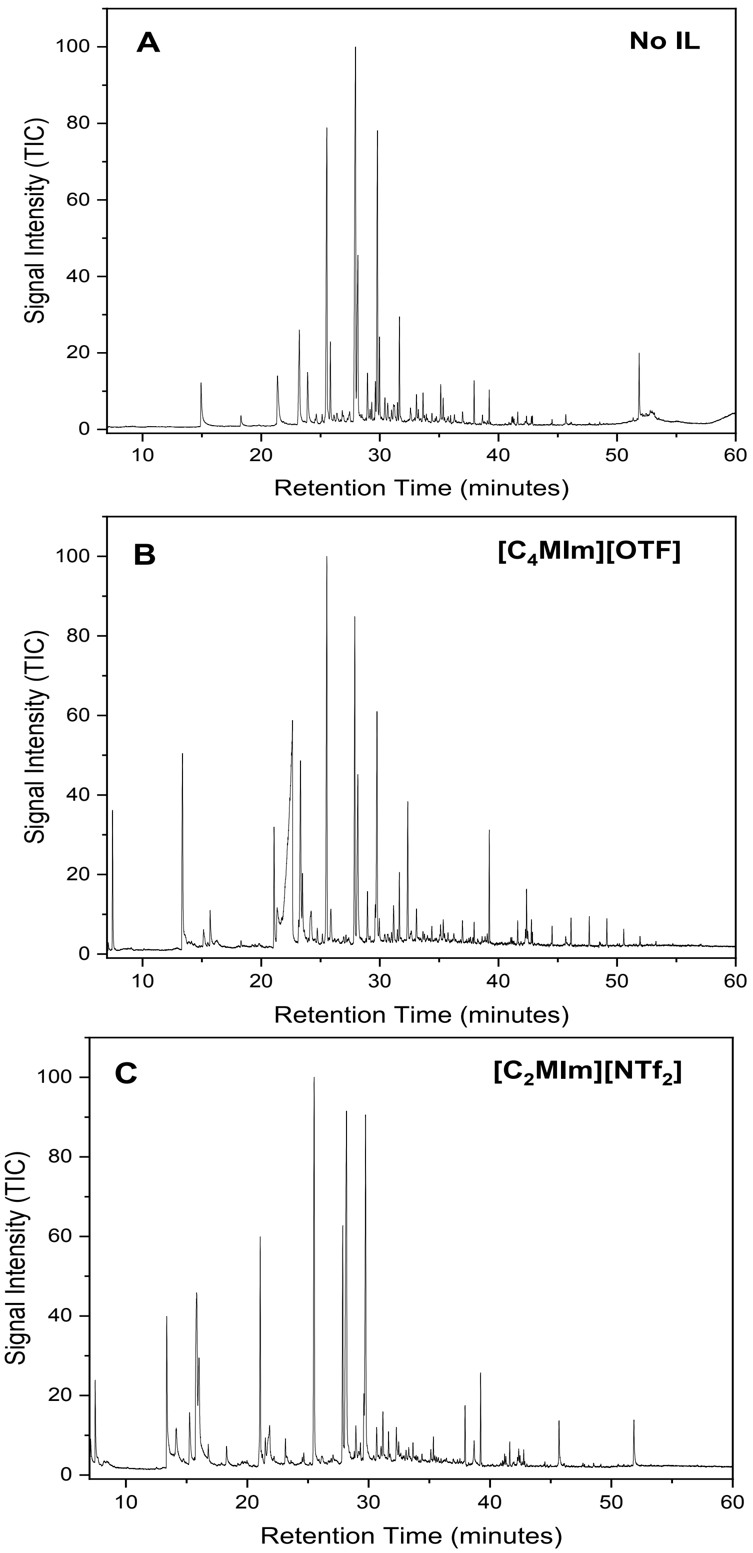

Figure S9. GC Chromatograms of tars produced from co-pyrolysis of lignin with **(A)** no IL, **(B)** $[C_4MIm][OTF]$, and **(C)** $[C_2MIm][NTF_2]$, respectively at 400 °C for 20 minutes.

Table S4. Distribution of tar products produced from co-pyrolysis of lignin with **(A)** no IL, **(B)** [C_4MIm][OTF], and **(C)** [C_2MIm][NTF_2], respectively at 400 °C for 20 minutes.

RT (min)	Compound Name	MW ($gmol^{-1}$)	Formula	No IL	[C_4MIm] [OTF]	[C_2MIm] [NTf_2]
13.4	1-methyl imidazole	82	$C_4H_6N_2$	-	+	+
14.9	Phenol	94	C_6H_6O	+	+	+
15.8	1-ethyl imidazole	96	$C_5H_8N_2$	-	+	+
18.3	2-methoxyphenol (from G-lignin)	124	$C_7H_8O_2$	+	+	+
21.1	Dodecane	170	$C_{12}H_{26}$	-	-	+
21.4	Catechol, 1,2-Benzenediol	110	$C_6H_6O_2$	+	-	-
22.6	1-Butylimidazole	124	$C_7H_{12}N_2$	-	+	-
23.2	3-methoxy-1,2-Benzenediol	140	$C_7H_8O_3$	+	+	-
23.9	4-methyl-1,2-Benzenediol	124	$C_7H_8O_2$	+	+	-
25.5	2,6-dimethoxyphenol (from S-lignin)	154	$C_8H_{10}O_3$	+	+	+
25.8	2,6-dimethoxyphenol (from S-lignin)	154	$C_8H_{10}O_3$	+	+	-
26.8	4-hydroxy-2-methoxybenzaldehyde	154	$C_8H_{10}O_3$	+	-	-
27.8	3,5-Dimethoxy-4-hydroxytoluene	168	$C_9H_{12}O_3$	+	+	+
28.1	4-Hydroxy-3-methylbenzoic acid	152	$C_8H_8O_3$	+	+	+
29.0	Apocynin	166	$C_9H_{10}O_3$	+	+	+
29.2	3,5-Dimethoxy-2,4-dimethylphenol	182	$C_{10}H_{14}O_3$	+	-	-
29.6	4-hydroxy-3-methoxy- methyl ester	182	$C_9H_{10}O_4$	+	+	+
29.7	4-hydroxy-3-methoxy- ethyl ester	177	$C_9H_{10}O_3$	-	-	+
29.8	Guaiacyl acetone	180	$C_{10}H_{12}O_3$	-	+	-
29.8	1,2,3-trimethoxy-5-methyl-toluene	182	$C_{10}H_{14}O_3$	+	-	-
30.0	Guaiacyl acetone	180	$C_{10}H_{12}O_3$	+	-	-
30.7	4-ethenyl-2,6-dimethoxy phenol	180	$C_{10}H_{12}O_3$	+	-	+
31.0	4-hydroxy-3-methoxy-methyl ester	196	$C_{10}H_{12}O_4$	+	-	-
31.2	1-(4-hydroxy-3-methoxyphenyl)- 1-propanone	196	$C_{10}H_{12}O_4$	+	+	-
31.2	4-hydroxy, 3-methoxy benzoic acid (Vanillic acid)	196	$C_{10}H_{12}O_4$	-	-	+
31.7	Homosyringaldehyde,	196	$C_{12}H_{19}NO_3$	+	+	+
32.6	(E)-2,6-Dimethoxy-4(prop-1-en-1-yl) phenol	194	$C_{11}H_{14}O_3$	+	+	-
34.3	1-(4-hydroxy-3,5-dimethoxyphenyl)-acetophenone	196	$C_{10}H_{12}O_4$	+	+	+
35.1	1-(4-hydroxy-3,5-dimethoxyphenyl)-acetophenone	196	$C_{10}H_{12}O_4$	+	+	-
38.0	Hexadecanoic acid, methyl ester palmitic acid	270	$C_{17}H_{34}O_2$	+	+	+
39.2	Hexadecanoic acid, ethyl ester palmitic acid	284	$C_{18}H_{36}O_2$	+	+	+
41.6	Methyl Stearate, Octadecanoic acid	298	$C_{19}H_{38}O_2$	+	+	+
42.3	Ethyl Oleate. Octadecanoic acid	310	$C_{20}H_{38}O_2$	+	+	+
42.8	Ethyl 15 methyl hexadecanoate	298	$C_{19}H_{38}O_2$	+	+	+
45.7	9-octadecenamide	281	$C_{18}H_{35}NO$	+	+	+
46.1	Eicosane	282	$C_{20}H_{42}$	+	+	-
47.6	Octacosane	394	$C_{28}H_{58}$	+	+	-
49.1	Octadecane	366	$C_{26}H_{54}$	+	+	-
51.9	Hexatriacontane	506	$C_{36}H_{74}$	-	+	-

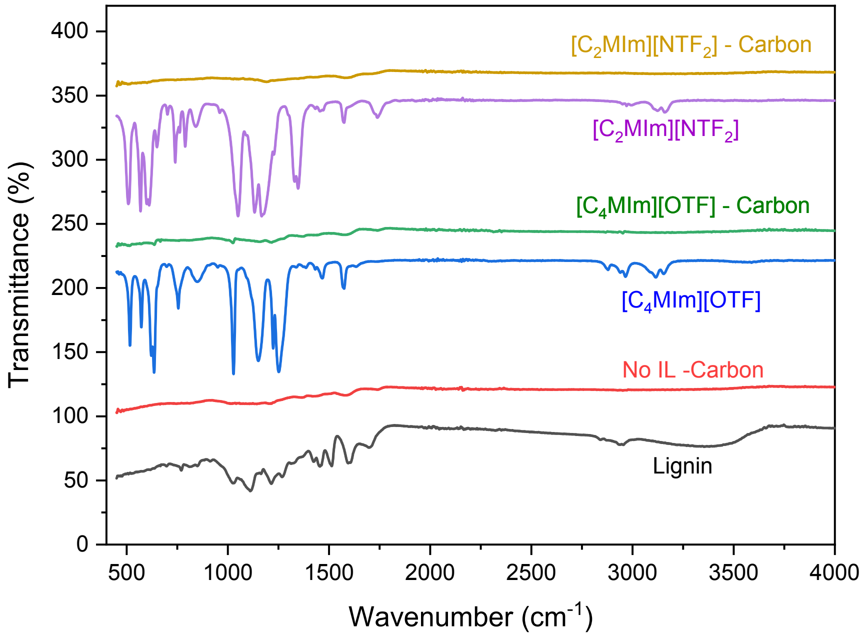

Figure S10. FT-IR spectra of lignin, [C_4MIm][OTF], [C_2MIm][NTF_2], lignin-derived carbons produced from co-pyrolysis of lignin with no IL, C_4MIm][OTF], and [C_2MIm][NTF_2], respectively at 400 °C for 20 minutes.

References

1. Anuchi, S. O., Campbell, K. L. S. & Hallett, J. P. Effective pretreatment of lignin-rich coconut wastes using a low-cost ionic liquid. *Sci Rep* **12**, (2022).

2. Kurnia, K. A., Lima, F., Cláudio, A. F. M., Coutinho, J. A. P. & Freire, M. G. Hydrogen-bond acidity of ionic liquids: an extended scale. *Physical Chemistry Chemical Physics* **17**, 18980–18990 (2015).

3. Park, T. J., Kim, Y. S., Kan, E. & Lee, S. H. Influence of ionic liquids as solvents for the chemical synthesis of poly(3-octylthiophene) with FeCl3. *RSC Adv* **5**, 25590–25593 (2015).

4. Jessop, P. G., Jessop, D. A., Fu, D. & Phan, L. Solvatochromic parameters for solvents of interest in green chemistry. *Green Chemistry* **14**, 1245–1259 (2012).

5. Tokuda, H., Hayamizu, K., Ishii, K., Susan, M. A. B. H. & Watanabe, M. Physicochemical properties and structures of room temperature ionic liquids. 2. variation of alkyl chain length in imidazolium cation. *Journal of Physical Chemistry B* **109**, 6103–6110 (2005).
